# Supplementary figures and images for: Perceptual prediction error supports implicit process in motor learning
Source: PLoS Comput Biol. 2026 Apr 15;22(4):e1014196. doi: 10.1371/journal.pcbi.1014196 (PMC13102310; doi:10.1371/journal.pcbi.1014196)

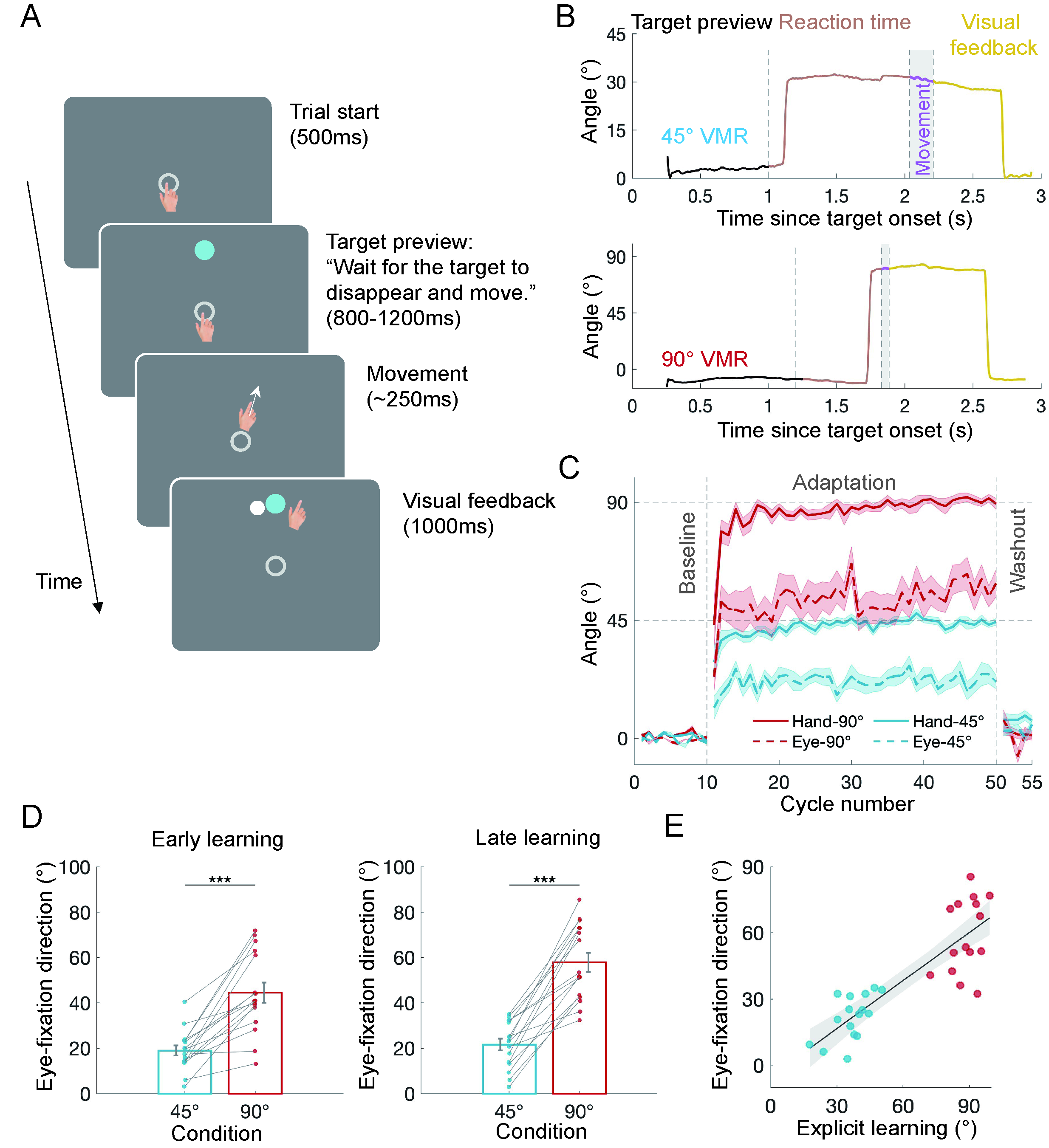

Supplement: S1 Fig — (A) Illustration of the experimental procedure. In each trial, participants first held their hand at the start position for 500 ms, awaiting the appearance of a target randomly positioned between 0° and 360°. They were instructed not to move until the target disappeared (~1000 ms after appearance). Visual feedback of the hand cursor was withheld until the trial ended, when it re-appeared together with the target as performance feedback. Eye tracking measurements were taken during reaching. (B) Time course of the gaze direction. Different colors represent different timings of a trial, including target preview, reaction time (RT), movement duration, and terminal feedback. Two representative trials from a participant are shown for the 45°-VMR (upper panel) and 90°-VMR (lower panel) conditions, respectively. The participant fixated on the starting position during the target preview and RT, then shifted fixation toward the re-aiming direction during the movement, maintaining this fixation until the terminal feedback was provided (purple line). The re-aiming direction was less rotated than the imposed VMR. After the movement ended and visual feedback was provided, participants tended to fixate back to the cursor (yellow line). We calculated the probability of fixations in the target area (± 22.5° around the target direction) after movement: 64.28 ± 4.91% in the 45°-VMR condition and 43.23 ± 6.80% in the 90°-VMR condition. (C) Average hand movement and eye fixation directions during adaptation to the 45° and 90° VMR. The fixation direction was defined as the last fixation before movement onset [41]. The shaded areas denote the standard errors across participants. From early learning, participants started to fixate away from the target direction, closer to their explicit re-aiming direction, and this fixation “strategy” persisted throughout the adaptation phase. Note that the fixation deviated further from the target in the 90°-VMR condition compared to the 45°-VMR conditio [file pcbi.1014196.s001.tif]

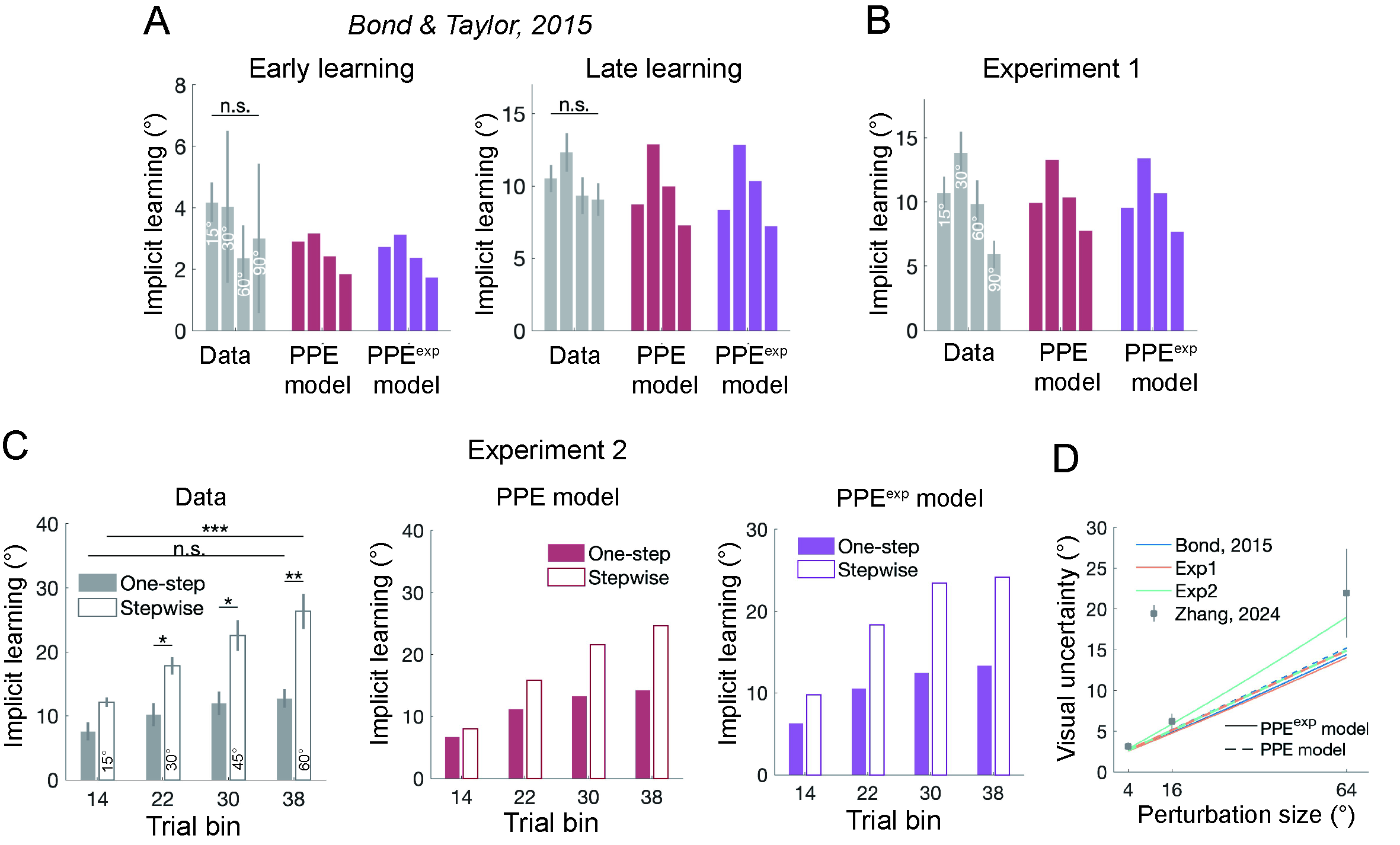

Supplement: S2 Fig — The observed and model-predicted implicit learning for Bond & Taylor 2015 (A), Experiment 1 (B), and Experiment 2 (C). The two versions of the PPE model similarly explain the data. (D) The visual uncertainty functions are estimated from the three datasets. Note the estimates are similar for the linear and the exponential functions, and they are also close to the visual uncertainty measured in our previous work [29]. (TIF) [file pcbi.1014196.s002.tif]

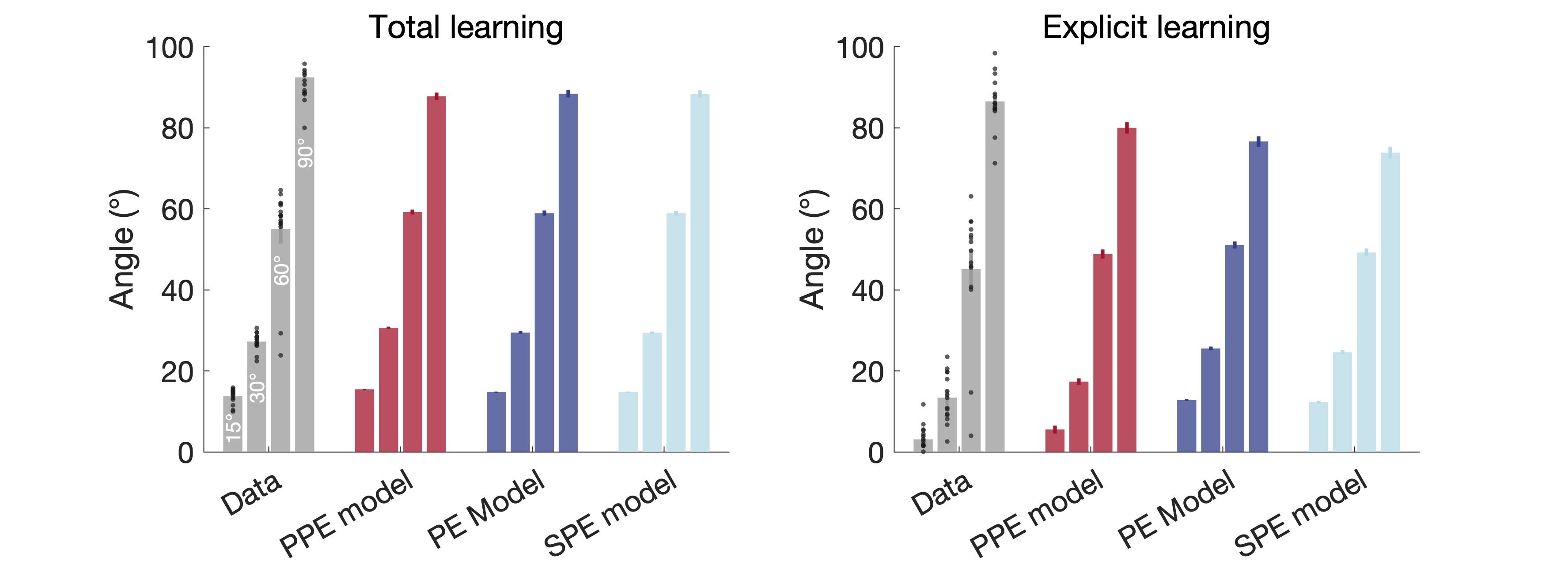

Supplement: S3 Fig — All three models reproduced the scaling effect of perturbation size for total and explicit learning. Error bars represent SEM for data and bootstrapped standard deviations for the model. The black dots represent individual data. (TIF) [file pcbi.1014196.s003.tif]

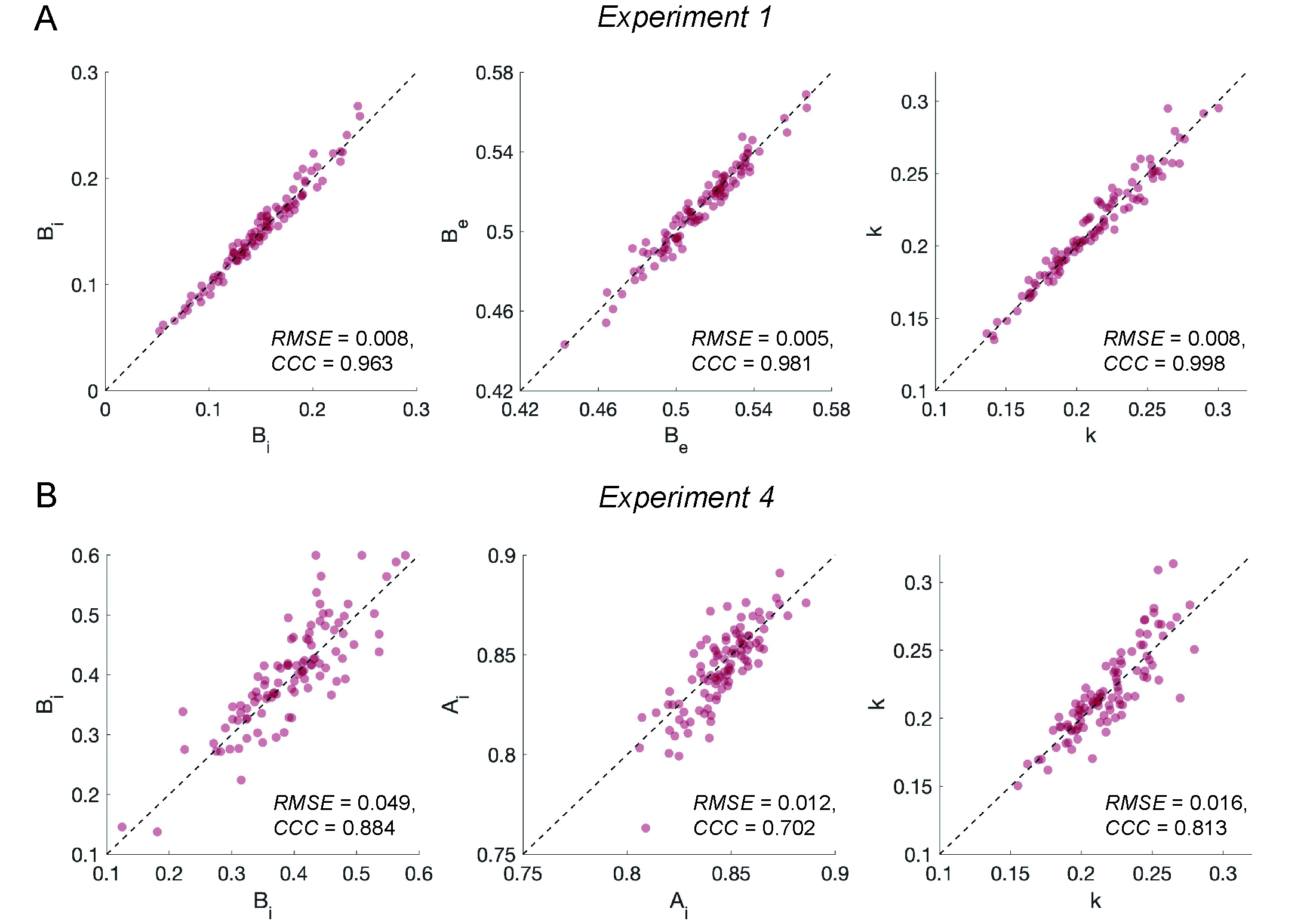

Supplement: S4 Fig — We chose Exp.1’s dataset to show the case when the model fits both explicit and implicit learning, and Exp.4’s to show the case when the model only fits implicit learning, as explicit learning is constrained by re-aiming instructions. For each experiment, we employed a bootstrap resampling approach (N = 5,000) to estimate the parameter distributions. We then used the median ± standard deviation of the bootstrapped parameters to define realistic parameter ranges for simulation. Critically, we incorporated motor noise into the simulations, estimated from the baseline movement variability of participants to ensure that our synthetic data reflected realistic noise characteristics. X-axis shows simulated parameters and y-axis shows recovered parameters. Simulated and recovered parameters showed high concordance, with concordance correlation coefficient (CCC) exceeding 0.98 for all three parameters in Exp.1 (A), and strong concordance with CCC exceeding 0.8 in Exp.4 (B). These results show that our fitting pipeline are reliable under realistic noise and sample sizes. (TIF) [file pcbi.1014196.s004.tif]

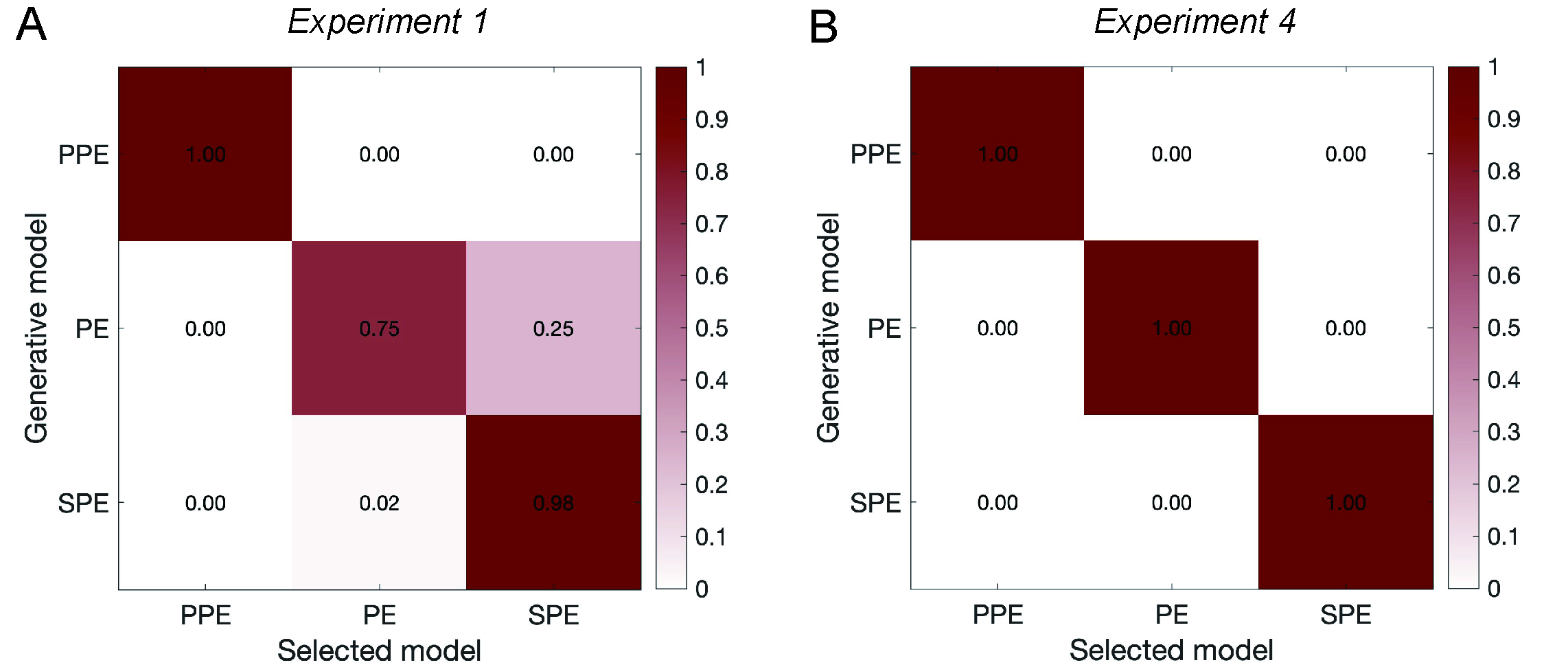

Supplement: S5 Fig — Confusion matrices of the three competing models (PPE, PE, and SPE models), using datasets from Exp.1 and Exp.4. We generated synthetic data from each generative model and applied our model selection procedure (the model with the lowest BIC) to these simulated datasets to assess whether the generative model could be correctly identified. The confusion matrices display the proportion of simulations in which each true generative model was recovered as the selected model. The results show strong discriminability among the three models. (TIF) [file pcbi.1014196.s005.tif]

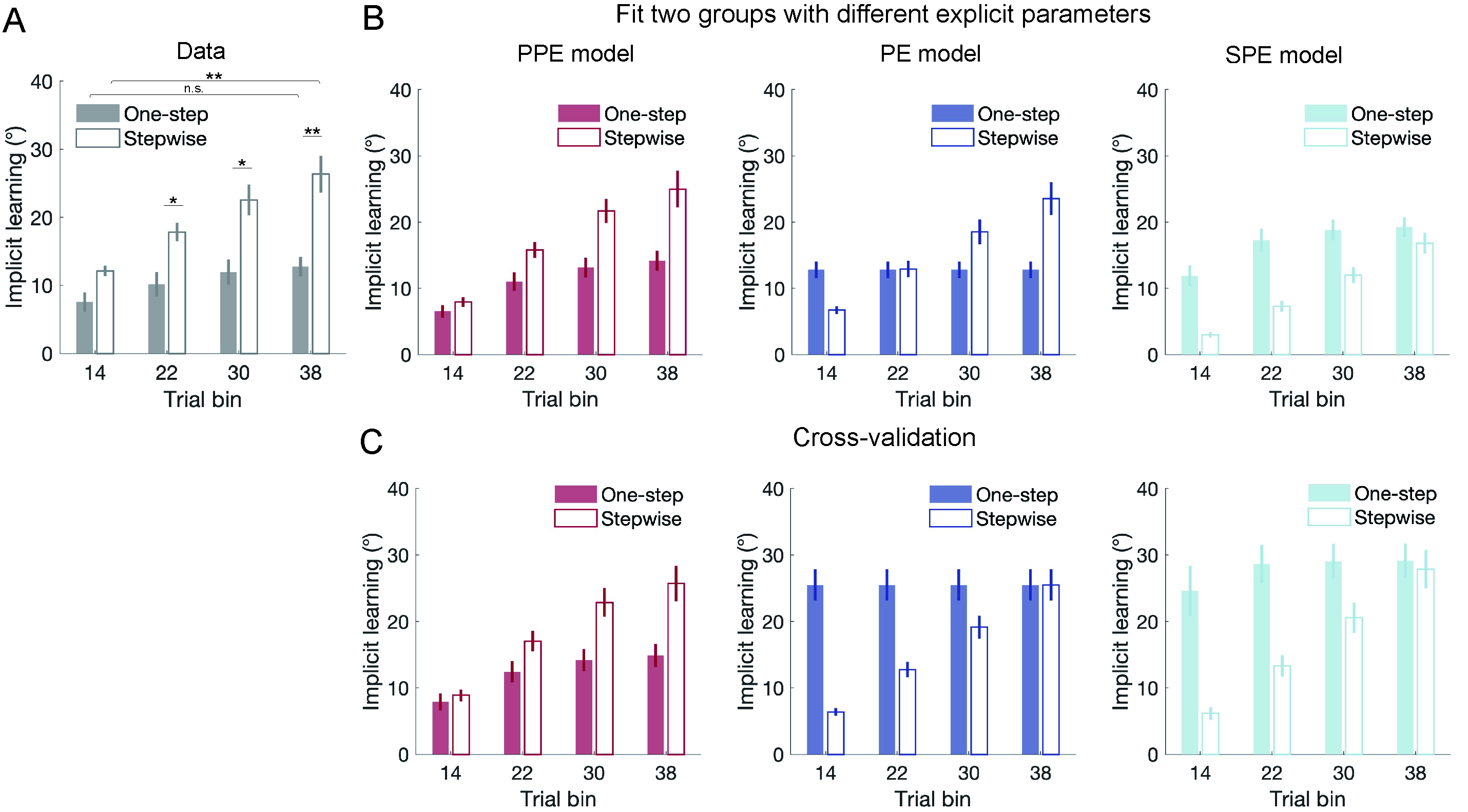

Supplement: S6 Fig — (A) Implicit learning data in Experiment 2, a reproduction of Fig 4C right panel. (B) Model fitting with varying explicit parameters across the two conditions. Compared to the model fitting in the main text (Fig 4D), here we allowed explicit parameters to vary across the two conditions. The PE and SPE models still failed to reproduce the data, especially the relative size of implicit learning between the two perturbation conditions. The PE model could not capture the implicit learning patterns for small stepwise perturbations (15° and 30°), and it yielded unrealistic model parameters (Table B in S3 Table). The SPE model showed minimal improvement, continuing to underestimate implicit learning in the stepwise condition and overestimate it in the one-step condition. The PPE model remained capable of explaining the data well with variable explicit learning parameters. These results suggest that the failure of PE and SPE to explain implicit learning across conditions was not due to an unconsidered potential parametric change in explicit learning. (C) Cross-validation tests for the three models. Each model was fit to the data from the stepwise condition and then used to predict implicit learning in the one-step condition. Neither PE nor SPE model captures the learning pattern: their predictions for the one-step group were highly inaccurate; both models overestimated implicit learning and underestimated explicit learning in the one-step condition. In contrast, the PPE model’s predictions match well with the empirical data across conditions, further reinforcing its robustness in capturing the dynamics of both implicit and explicit learning across different perturbation schedules. In (B) and (C), error bars represent bootstrapped standard deviations (resample size = 5,000). (TIF) [file pcbi.1014196.s006.tif]

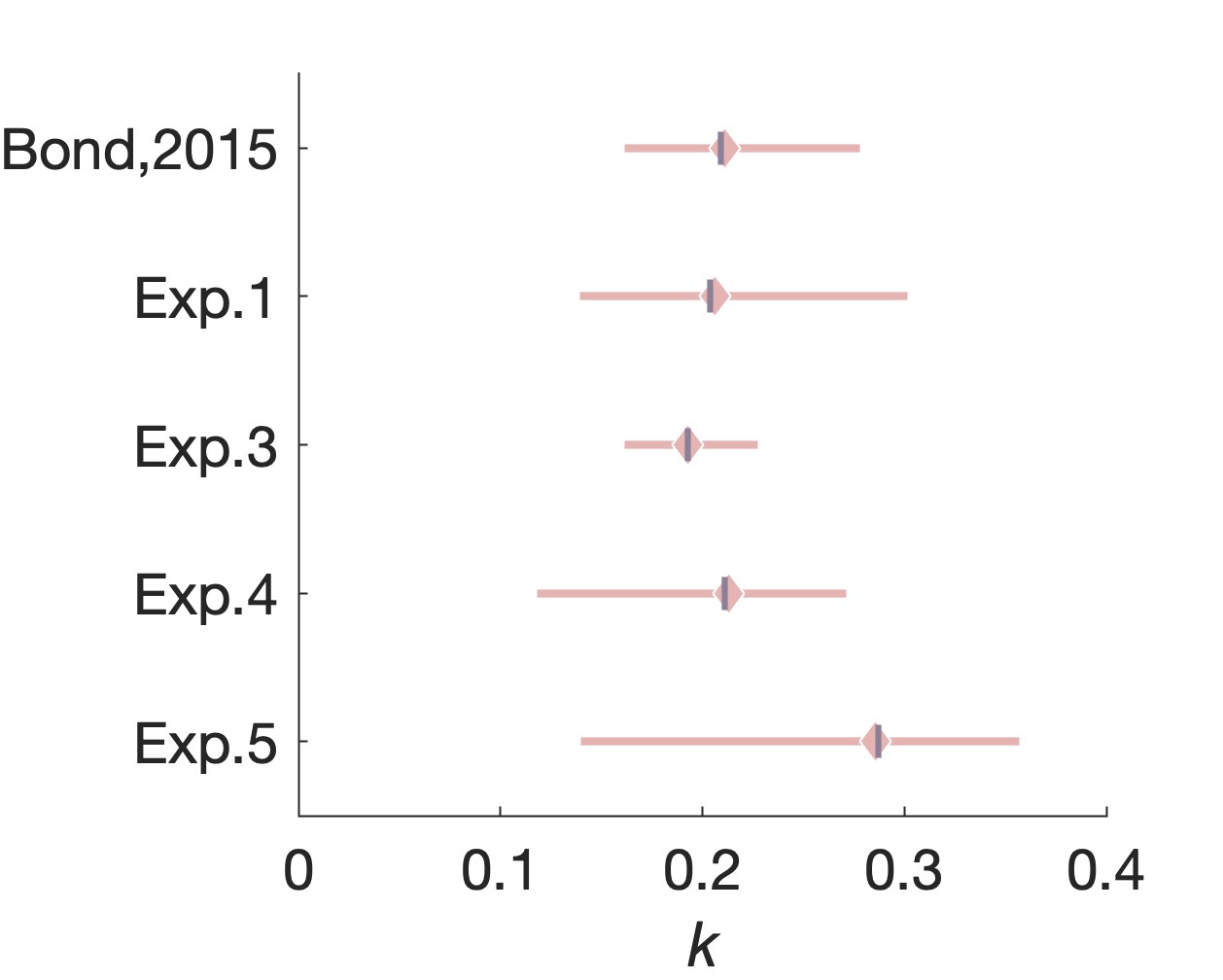

Supplement: S7 Fig — Pink diamond markers indicate median values estimated using bootstrap resampling (N = 5,000), with horizontal error bars representing 95% confidence intervals. Purple line markers represent parameter values estimated by fitting the average learning data, which closely align with the bootstrap median estimates, demonstrating robust and consistent model fits across experiments. (TIF) [file pcbi.1014196.s007.tif]

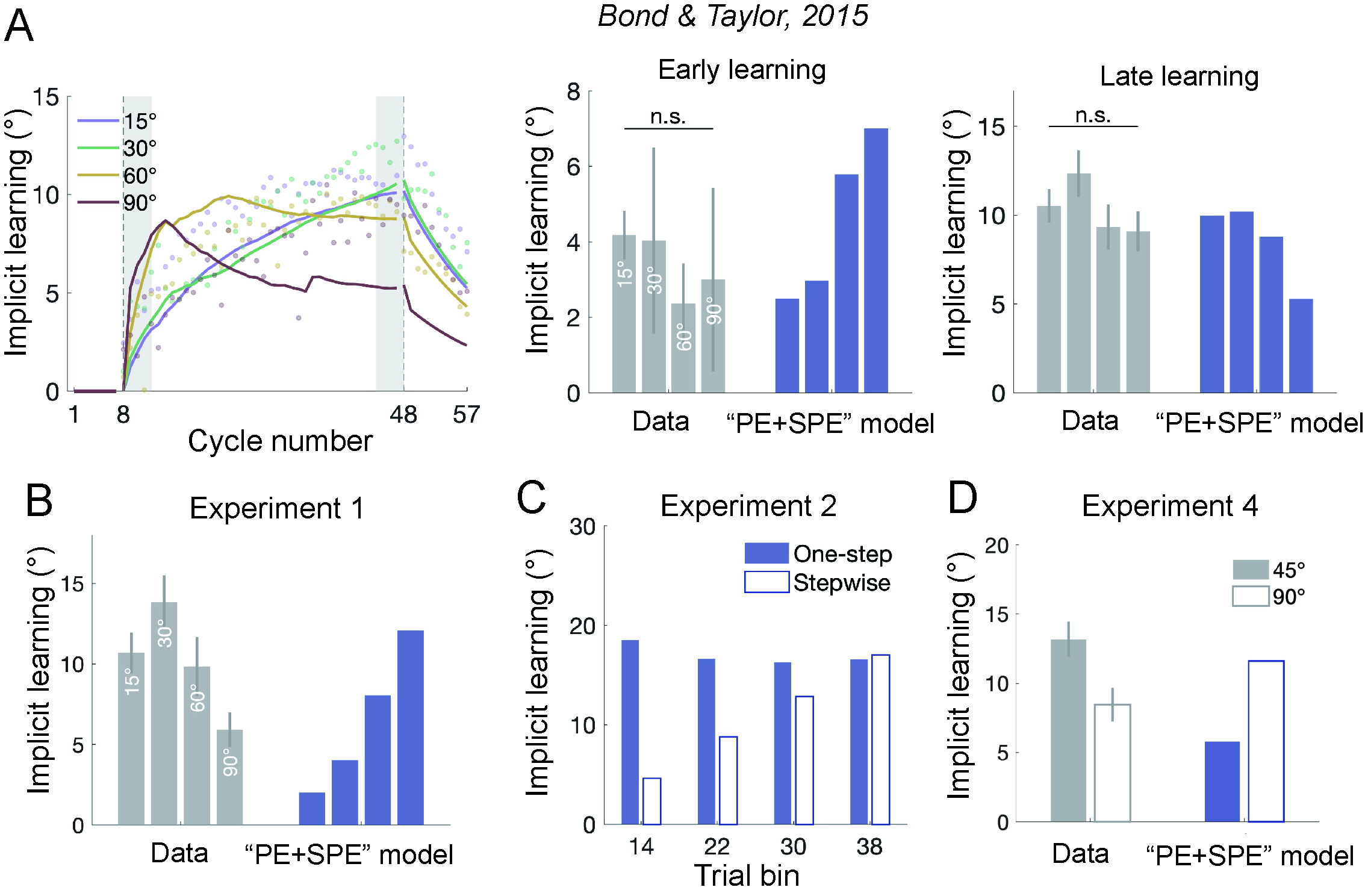

Supplement: S8 Fig — We used different datasets to test the model: (A) Bond & Taylor, 2015; (B-D) Our Experiments 1, 2, and 4. The model analysis showed that the two-state model failed to explain the data. Furthermore, parameter estimates (a very small B) from the model fitting suggest that incorporating an additional implicit learning component is unnecessary (S8 Table). (TIF) [file pcbi.1014196.s008.tif]

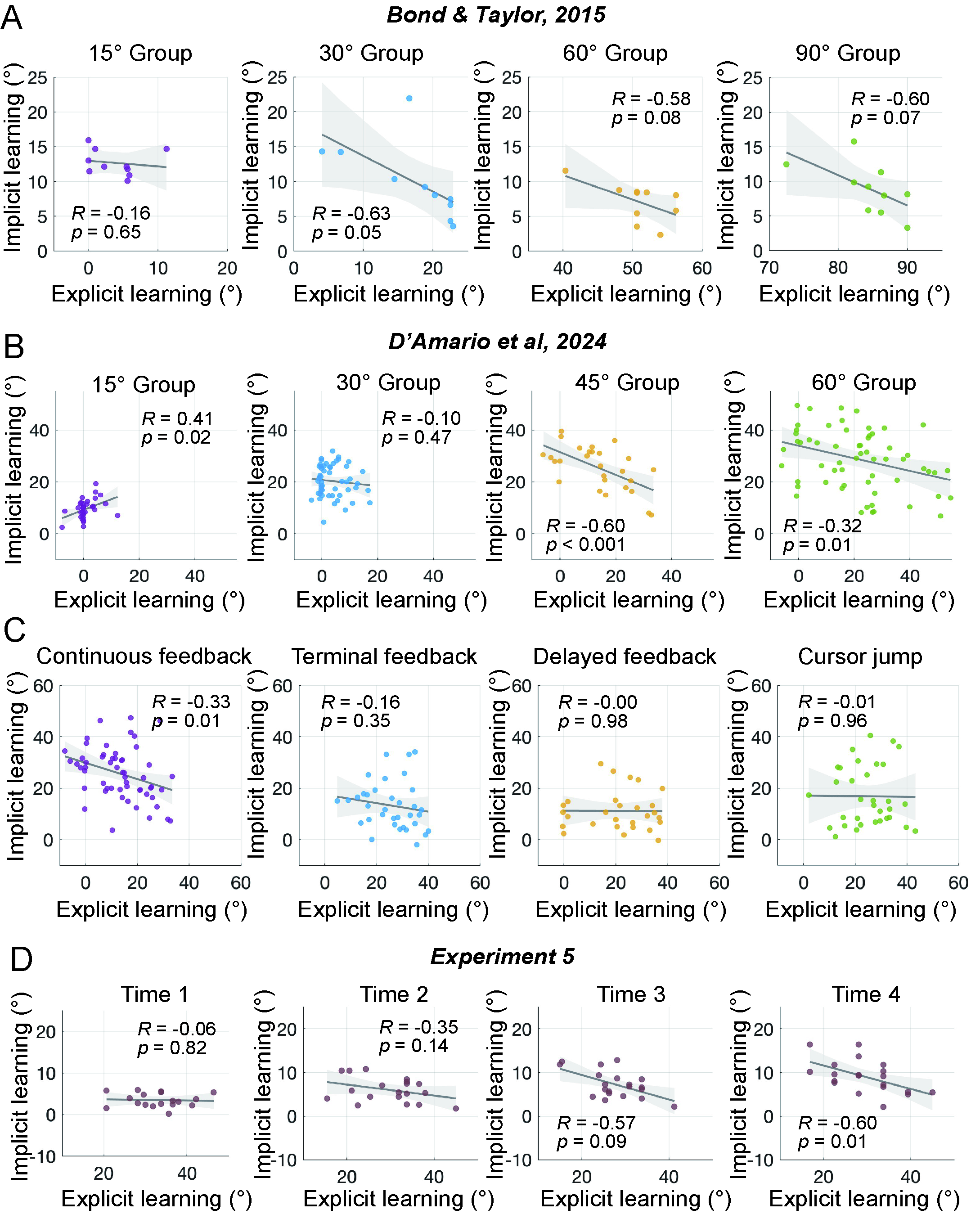

Supplement: S9 Fig — Data from three separate studies are presented, with explicit learning measured by aiming reports and implicit learning measured by no-feedback exclusion trials. (A) Correlations between explicit and implicit learning in different perturbation size conditions, including 15°, 30°, 60°, and 90° VMR (n = 10 for each). Due to the small sample size and variability across conditions, support for a negative correlation was insufficient. Data from Bond & Taylor, 2015. (B) Correlations across perturbation size conditions of 15°, 30°, 45°, and 60° VMR (n = 33, 54, 28, 61, respectively) from D’Amario et al., 2024. With decent sample sizes, positive, negative, or null correlation was observed in these different conditions. (C) Correlations were inconsistent when the format of visual perturbation varied, but with a fixed perturbation size of 45° VMR. Data from D’Amario et al., 2024. Continuous cursor feedback (n = 51) is the conventional feedback format, terminal feedback promotes explicit learning and reduces implicit learning (n = 35), delayed feedback reduces implicit learning (n = 39), and cursor jump feedback usually promotes explicit learning and reduces implicit learning (n = 32). Only the continuous-feedback condition showed a negative correlation; no correlation was detected for the other three feedback formats. (D) Early learning did not show a negative correlation. In our Experiment 5 (n = 20), explicit learning and implicit learning were independently measured four times during the 60-trial-cycle learning period. The two early instances (the 5th and 19th cycles) did not show significant negative correlations, the third instance (the 33rd cycle) showed a marginal effect, and the fourth instance (the 47th cycle) showed a significant negative correlation. Thus, though performance error is most prominent during early learning, there is no evidence that the two learning processes compete for this error and then lead to a negative correlation. Each dot represents an indivi [file pcbi.1014196.s009.tif]
