# Supplementary material for: Perceptual prediction error supports implicit process in motor learning
Source: PLoS Comput Biol. 2026 Apr 15;22(4):e1014196. doi: 10.1371/journal.pcbi.1014196 (PMC13102310; doi:10.1371/journal.pcbi.1014196)
Supplement: Attachment — Submitted filename: Response to Reviewers.docx [file pcbi.1014196.s019.docx]

Reviewer #1: In this paper, Zhang et al. continue to explore the recent perceptual prediction error ("PPE") model of implicit motor adaptation. To do this, they both re-analyze previously published datasets, and also performed several new experiments to pit the PPE model against other prominent ideas about the error signals that drive motor learning, the sensory prediction error (SPE) idea, and the performance error (PE) idea. Their experiments involve novel behavioral manipulations, as well as computational modeling, proprioceptive assays, and eye-tracking. Overall, the authors argue that the data best support the PPE model of motor adaptation, which involves a Bayesian weighting of proprioception and vision to estimate hand position given a desired movement and compare it to observations, and argue strongly against the SPE and PE models.

Even though the model presented here has been previously outlined, this study represents a rigorous and expanded testing of that model in a series of novel and impressive experiments and modeling analyses. In general, I read this article with great enthusiasm --- by the end of it, I was rather convinced that the authors' PPE model is indeed the current best model of implicit motor adaptation. My critiques are mostly minor. I applaud the authors on a very thorough and rigorous set of experiments.

We would like to express our appreciation of your encouraging and constructive comments. We followed your suggestions point-by-point and substantially improved our paper.

Strengths:
- the study uses models to make clear predictions, and clever experiments to test those predictions. Overall, the results are rather impressive and lend rather strong support to the PPE model
- the figures are clear and nicely detailed
- the methods are clear and the range of control analyses really adds to the robustness of the modeling
- the eye-tracking experiment is a very nice touch
- the broader implications of the study are nicely laid out in the Discussion, especially w/r/t other forms of motor learning (e.g., speech).


Weaknesses:

- i was somewhat confused about the implied framing of SPE as not being "perceptual"; it seems that an SPE model can easily assume that the visual error is routed through the brain's perceptual computations, not just some kind of direct sensory readout (whatever that would be anyway). the nomenclature used thus makes the nature of the SPE model seem somewhat odd. isn't the main advance here that the prediction errors that drive implicit motor learning involve an uncertainty-weighted perceptual estimate of the hand, not just the visual error? Isn't that what primarily differentiating SPE and PPE, rather than "sensation versus perception"?

Thank you for raising this nomenclature issue. We completely agree that the more specific distinction for VMR is between perceptual and visual estimation. SPE and PPE are both prediction errors. Notably, they share the same prediction, i.e., both assume that the prediction is about where the hand intends to be (aiming direction for VMR), but they differ in the definition of feedback: SPE involves the feedback about where the visual cursor actually goes, whereas PPE uses the perceived hand direction, resulting from multisensory integration, as feedback. Visual feedback is unimodal, but the perceptual estimate is multimodal, integrating multiple cues weighted by their uncertainty. For perceptual estimation, these cues are not restricted to sensory information; in our framework, motor prediction (intended direction) is included for perceptual estimate. In Bayesian multisensory integration, motor prediction can be viewed as a prior, as it is available before movement. In fact, a recent study has shown that, even without actual movement, motor prediction or motor planning that deviates from the target can still elicit implicit adaptation (Kim et al., 2022), a finding consistent with our conceptualization of PPE and its inclusion of motor prediction.

We now elaborate on the terminology about sensory vs perceptual prediction error in the Discussion to make our idea clear: “Our study provides strong evidence that visual prediction error (typically named as SPE) cannot adequately capture the dynamics of implicit learning, even when the perturbation itself is visual. In contrast, perceptual prediction error (PPE), which is derived from multisensory integration, can parsimoniously explain both our data and key findings in the literature (15, 34, 53). Notably, PPE and SPE share the same prediction, i.e., both assume that the prediction is about where the *hand* intends to be (aiming direction for VMR). However, they differ in the definition of feedback: SPE uses the visual cursor as feedback to compare with the hand prediction, while PPE uses the perceived hand direction (resulting from multisensory integration) as feedback. Interestingly, prediction and feedback for SPE do not refer to the same entity (hand vs. cursor), whereas for PPE, they both refer to hand direction. The term “perceptual” in PPE highlights the fact that the feedback used to compute this prediction error is a percept, a posterior estimate of effector localization in Bayesian terms.”

- there could be more caveats offered for why the PPE model fit in Figure 2C is not particularly strong. i understand that it clearly outperforms the SPE and PE models; but why is it not a better fit here? this is a small point as the fit is better in the later experiments, but still warrants some discussion.

Thanks for this detailed observation. Indeed, Figure 2C, compared to Figure 3, appears to suggest that the PPE model explains less variance in the data. This is also reflected in the smaller *R^2^* values (0.708 vs. 0.969), even though the PPE model clearly outperforms the SPE and PE models. This is due to the specific characteristics of the *Bond & Taylor, 2015* dataset. For this dataset, we fit implicit learning data, which was derived by subtracting explicit learning (measured by aiming reports) from total learning (hand direction). The aiming reports exhibited considerable variability, both within and across subjects. The large variance in aiming reports leads to substantial noise in the estimates of implicit learning. As can be observed in the data, the implicit learning data is noticeably more variable in the Bond & Taylor dataset than in our own experiment 2 (Figure 2C vs. Figure 3A). Since implicit learning data constitute the majority of data for model fitting, thus its higher variance naturally limits the achievable fitting quality for all models. This reduction in *R^2^* does not reflect a limitation of the PPE model but rather the inherent noise in the data being fitted.

- there are some data with rather low asymptotic implicit learning (~10 degrees or so). why is this the case? considerations of experimental apparatus lag, or temporally volatile adaptation, could be offered to address this (see recent papers by Hadjiosif et al on these two issues). could it be that there are different aspects of implicit adaptation (e.g., volatile and stable) that have different error sources?

Thanks for raising the issue of implicit learning magnitude. The questions appear to be twofold: First, whether our reported implicit learning in some experiments is relatively small (~10 degrees) and whether this is related to apparatus lag or the delay (related decay of implicit learning raised by Hadjiosif and colleagues’ recent work). Second, whether this relates to multiple states of implicit learning with different time scales. We will elaborate on these two issues separately below.

First, the asymptotic implicit learning reported here is in the normal range when compared to similar experiments in the literature. Implicit learning of ~10° has been reported widely in the VMR paradigm (Albert et al., 2022; Taylor et al., 2014; Bond & Taylor, 2015; McDougle et al., 2017; Neville & Cressman, 2018; Tsay et al., 2021). The magnitude of implicit learning depends on experimental manipulations and measurements. For example, large rotation size leads to low implicit learning (shown in Bond & Taylor, 2015; Tsay et al., 2021; our own Exp.1). The enhancement of explicit learning, through instruction or frequent aiming reports, also leads to low implicit learning: Bond & Taylor (2015) had participants report their aiming direction on each trial, encouraging the strategy adoption (see also Maresch et al., 2021), leading to a relatively low implicit learning. This is also shown in our Exp. 5, where we conducted interleaved aiming reports. In fact, the perturbation schedule also affects implicit learning: abrupt perturbations lead to smaller implicit learning than stepwise perturbations. Our Experiment 2 showed implicit learning increased to more than 25° in the stepwise condition compared to ~10° in the abrupt condition. Thus, we believe the implicit learning magnitudes reported here span a wide range, but they are all within the normal range expected from the literature. Furthermore, the variation in asymptotic implicit learning reflects task-specific factors, not apparatus lag or delay. Our setups are used across experiments and the large variations in implicit learning across experiments are dominated by their task-specific factors.

Second, regarding multiple components of implicit adaptation, previous research has indeed suggested that implicit adaptation may comprise two components with fast and slow time scales (Hadjiosif et al., 2023). The slow and fast time-scale components are dissociable during inserted rest periods. Note, our experimental paradigms did not insert rest intervals, which we believe makes isolation of the two components with different time scales challenging. Nevertheless, in the revision, we constructed an implicit learning model with fast and slow components driven by different errors. Based on the literature, we assumed that the fast one is driven by performance error and the slow one by sensory prediction error. However, we found that this two-state model failed to explain the major findings of all the key experiments here (Figure R1; Table R1). Of course, these results do not argue against the possibility that implicit learning contains multiple components with different time scales. They merely suggest that error-based implicit adaptation is not driven by two visual errors operating at different time scales. Using PPE to explain the two states of implicit adaptation appears to be an interesting research question for future studies.

We have now discussed the possibility of two time scales in the revised Discussion: ” We also considered the possibility that implicit learning is driven by sensory prediction error and performance error simultaneously. Previous research has suggested that implicit adaptation may comprise a fast and a slow component (70). We constructed an alternative model where implicit learning’s fast and slow components are driven by performance error and sensory prediction error, respectively. However, we found that this two-implicit-state model failed to explain the major findings of the key experiments here (Figure S8; Table S8). We want to emphasize that these results do not argue against the possibility that implicit learning contains multiple components with different time scales. Rather, they suggest that the two visual errors, i.e., SPE and PE, are unable to drive the two time-scale components for implicit adaptation. Future studies are needed to examine whether perceptual prediction error identified here can adequately model the potential fast and slow components in implicit adaptation.”

**Figure R1.** Model analysis using a two-state model for implicit learning, which assumes that implicit learning comprises fast and slow components driven by both performance error (PE) and sensory prediction error (SPE), respectively. We used different datasets to test the model: **(A)** Bond & Taylor, 2015; **(B-D)** Our Experiments 1, 2, and 4. The model analysis showed that the two-state model could not well explain the data. Furthermore, parameter estimates (a very small *B*) from the model fitting suggest that incorporating an additional implicit learning component is unnecessary (Table R1).

**Table R1.1** Model comparisons and parameters for the dataset from *Bond & Taylor, 2015*

|  | *R^2^* | *RMSE(°)* | AIC | BIC | *B_i_* | *A_i_* | *B_i-PE_* | *A_i-PE_* |
| --- | --- | --- | --- | --- | --- | --- | --- | --- |
| PPE Model | **0.708** | **1.684** | **172.81** | **182.04** | 0.158 | 0.931 | / | / |
| Two-state Model | 0.093 | 2.244 | 266.65 | 278.95 | 0.069 | 0.999 | 0.010 | 0.010 |

Note, in the tables, *B_i_* and *A_i_* denote parameters of the SPE/PPE-driven implicit learning component, while *B_i-PE_* and *A_i-PE_* denote parameters of the PE-driven implicit learning component for the two-state model.

**Table R1.2** Model comparisons and parameters for the data from Experiment 1

|  | *R^2^* | *RMSE(°)* | AIC | BIC | *B_i_* | *A_i_* | *B_i-PE_* | *A_i-PE_* | *B_e_* | *A_e_* |
| --- | --- | --- | --- | --- | --- | --- | --- | --- | --- | --- |
| PPE Model | 0.969 | 4.807 | 359.67 | 370.55 | 0.151 | 0.981 | / | / | 0.513 | 0.981 |
| Two-state Model | 0.956 | 5.902 | 409.65 | 425.96 | 0.003 | 0.988 | 0.053 | 0.988 | 0.463 | 0.988 |

**Table R1.3** Model comparisons and parameters for the data from Experiment 2

|  | *R^2^* | *RMSE(°)* | AIC | BIC | *B_i_* | *A_i_* | *B_i-PE_* | *A_i-PE_* | *B_e_* | *A_e_* |
| --- | --- | --- | --- | --- | --- | --- | --- | --- | --- | --- |
| PPE Model | **0.960** | **3.965** | **252.43** | **264.82** | 0.245 | 0.927 | / | / | 0.889 | 0.927 |
| Two-state Model | 0.933 | 5.236 | 303.38 | 318.25 | 0.010 | 0.967 | 0.330 | 0.896 | 0.570 | 0.967 |

**Table R1.4** Model comparisons and parameters for the data from Experiment 4

|  | *R^2^* | *RMSE (°)* | *AIC* | *BIC* | *B_i_* | *A_i_* | *B_i-PE_* | *A_i-PE_* |
| --- | --- | --- | --- | --- | --- | --- | --- | --- |
| PPE Model | **0.972** | **0.524** | **-32.81** | **-28.61** | 0.396 | 0.840 | / | / |
| Two-state Model | -1.187 | 4.456 | 97.65 | 103.26 | 0.209 | 0.974 | 0.027 | 0.999 |

- as a discussion point, i'm curious what the authors think about the fact that deafferented patients can show somewhat normal implicit adaptation (Tsay et al., 2024). this seems like an interesting mystery.

Thank you for directing us to the seemingly conflicting but interesting findings. The deafferented patients, with their impaired proprioceptive feedback, showed normal implicit adaptation in Tsay et al.’s (2024) study, while our model should predict an increase in implicit adaptation with increasing proprioceptive uncertainty IF all other factors remain the same. However, deafferented patients exhibited compensatory changes for their proprioceptive loss in both perception and action. For example, they appear to rely exclusively on the efference-based central sense of effort to judge force and weight (Lafargue et al., 2003). They also preserved and even exaggerated their anticipatory grip-force scaling and predictive strategies for object manipulation, while their feedback corrections are impaired (Hermsdörfer et al., 2008). These results appear to support that they re-weight efference-related or predictive cues for sensorimotor control after lifelong compensation. We thus believe that normal implicit adaptation in deafferented patients might be related to their re-weighting of sensory cues (visual and proprioceptive) and motor prediction. This hypothesis can be tested by measuring PPE indirectly (using the passive localization tests in our Experiments 1 and 2) or directly (our Experiment 6).

Furthermore, we would like to emphasize that while findings from deafferented patients (e.g., Tsay et al., 2024) are valuable and informative, inferences drawn from comparisons with healthy individuals should be interpreted cautiously due to long-term adaptation and compensation in these patients. The critical test of our model, or the more direct and less ambiguous one, should be short-term manipulations in healthy participants. Taking proprioception as an example, our unpublished experiments directly manipulated proprioceptive uncertainty in healthy participants by simply requiring them to implicitly adapt to visual perturbations in different regions of the workspace. We focused on the fact that people’s proprioceptive sense is more uncertain when their hand is farther away from the body (which we confirmed psychophysically). Indeed, just as our PPE model predicted, participants showed exaggerated implicit learning when their movements were farther from the body compared to closer to the body.

We have added our interpretation of deafferented patients and proprioceptive uncertainty in our revised manuscript: “One interesting finding regarding the role of proprioception in implicit adaptation is that the deafferent patients, surprisingly, exhibited normal implicit adaptation (120). This appears contradictory to our PPE model predictions: the increase in proprioceptive uncertainty among deafferented patients should increase PPE and thus enhance implicit learning if all other factors remain the same. However, deafferented patients undergo lifelong compensatory changes for their proprioceptive loss in both perception and action. For instance, they relied exclusively on efference-based central sense of effort to judge force and weight (121), and they preserved and even exaggerated their anticipatory grip-force scaling and predictive strategies for object manipulation, while their feedback corrections are impaired (122). These findings suggested that they re-weight efference-related or predictive cues for sensorimotor control after lifelong compensation. We thus postulate that the observed intact implicit adaptation among deafferented patients might be related to their re-weighting of sensory cues and predictive cues. This hypothesis can be tested by measuring PPE indirectly (using the passive localization tests in our Experiments 1 and 2) or directly (our Experiment 6) among them in future investigations.”

Minor points:

- the legend at the top right of figure 4B has incorrect color labels
- the dark versus light gray shadings in figure 4A are hard to distinguish

Thank you. We fixed Figure 4.

- the instructions noted in figure 5 to "make the cursor hit the target" are confusing; don't the subjects not yet know the rotation sign/magnitude on such trials? how would they interpret that instruction?

We agree that the instructions may seem confusing given the random nature of the perturbations, but we used these instructions to emphasize the task-relevance of visual feedback, as consideration of the cursor’s task-relevance would affect adaptive learning on subsequent trials. We want to emphasize that throughout the current study, we used the VMR paradigm in which the cursor is always task-relevant. Although the unpredictability of the cursor regarding rotation sign and magnitude makes the task challenging, goal-directed actions require specific instructions about the task goal, i.e., emphasizing the cursor’s role in achieving the task.

Practically, this instruction will not change the aim on perturbation trials. Because the perturbations are random and balanced in sign, the aiming direction is on average straight toward the target. Moreover, given the emphasis on fast and straight movements, we expect no feedback-based corrections. Indeed, we found that the average movement direction was toward the target (1.16° ± 0.76°).

Similar instructions have been used previously in random perturbation experiments (e.g., Hutter & Taylor, 2018). They used a similar random perturbation paradigm with instructions to hit the target with the cursor. Their results also showed that participants’ average explicit learning approached zero, i.e., they aimed for the target. In sum, although the task-relevant instruction regarding the cursor makes the task challenging, previous studies and our own experiment have shown that participants can fulfil the task without any re-aiming strategy. More importantly, participants implicitly adapted to the errors induced by this special design, which provided us the opportunity to disambiguate the roles of three potential motor errors.

We have now clarified the rationale for this experimental design and instructions in the Method section of our revised manuscript: “Of note, despite the randomness of the perturbations, we emphasize to participants that the cursor is task-relevant and they need to use the cursor to hit the target despite the perturbation. Although this task-relevant instruction could make the task challenging, it emphasizes the task-relevance of visual feedback, which could affect adaptive learning. A previous study has shown that under these instructions and with random perturbations, participants can fulfil the task without any re-aiming strategy (136).”

- it would be nice to see a histogram of k values across all experiments, or bar graphs for each experiment.

Thanks for your suggestion. We have now included a forest plot in the Appendix (Figure R2/S7) to visualize the parameter estimates (*k*) across experiments. In this figure, the pink diamond markers indicate median values estimated using bootstrap resampling (N = 5,000), with horizontal error bars representing 95% confidence intervals. Importantly, the parameter *k* exhibited high consistency across different experiments, with similar median values and overlapping confidence intervals.

We also display parameter values estimated from fitting the average learning data (purple line markers). Note that these group-level estimates of *k* closely align with the bootstrap estimates, suggesting that our model fitting procedure has decent reliability and internal consistency.

In the revision, we have also provided the 95% confidence intervals of all the parameter estimates in Tables S1-S6 in the Appendix.

**Figure R2**. Estimated values of the parameter *k* (the slope of visual uncertainty as a function of visual eccentricity) of the PPE model across different experiments. Pink diamond markers indicate median values estimated using bootstrap resampling (N = 5,000), with horizontal error bars representing 95% confidence intervals. Purple line markers represent parameter values estimated by fitting the average learning data, which closely align with the bootstrap median estimates, demonstrating robust and consistent model fitting across experiments.

Note that we did not include Experiment 2 in this figure, because for the one-step group in Experiment 2, we adopted the *k* value estimated from Experiment 1, which employed identical abrupt perturbations.

**References**

Albert, S. T., Jang, J., Modchalingam, S., ’t Hart, B. M., Henriques, D., Lerner, G., Della-Maggiore, V., Haith, A. M., Krakauer, J. W., & Shadmehr, R. (2022). Competition between parallel sensorimotor learning systems. *eLife*, *11*. https://doi.org/10.7554/eLife.65361

Bond, K. M., & Taylor, J. A. (2015). Flexible explicit but rigid implicit learning in a visuomotor adaptation task. *Journal of Neurophysiology*, *113*(10), 3836–3849.

Hadjiosif, A. M., Morehead, J. R., & Smith, M. A. (2023). A double dissociation between savings and long-term memory in motor learning. *PLoS Biology*, *21*(4), e3001799.

Hermsdörfer, J., Elias, Z., Cole, J. D., Quaney, B. M., & Nowak, D. A. (2008). Preserved and impaired aspects of feed-forward grip force control after chronic somatosensory deafferentation. *Neurorehabilitation and neural repair*, *22*(4), 374-384.

Hutter, S. A., & Taylor, J. A. (2018). Relative sensitivity of explicit reaiming and implicit motor adaptation. Journal of Neurophysiology, 120(5), 2640–2648.

Kim, H. E., Parvin, D. E., & Ivry, R. B. (2019). The influence of task outcome on implicit motor learning. *eLife*, *8*(e39882).

Kim, O. A., Forrence, A. D., & McDougle, S. D. (2022). Motor learning without movement. *Proceedings of the National Academy of Sciences*, *119*(30), e2204379119.

Lafargue, G., Paillard, J., Lamarre, Y., & Sirigu, A. (2003). Production and perception of grip force without proprioception: is there a sense of effort in deafferented subjects?. *European Journal of Neuroscience*, *17*(12), 2741-2749.

Maresch, J., Werner, S., & Donchin, O. (2021). Methods matter: Your measures of explicit and implicit processes in visuomotor adaptation affect your results. *The European Journal of Neuroscience*, *53*(2), 504–518.

McDougle, S. D., Bond, K. M., & Taylor, J. A. (2017). Implications of plan-based generalization in sensorimotor adaptation. *Journal of Neurophysiology*, *118*(1), 383–393.

Neville, K.-M., & Cressman, E. K. (2018). The influence of awareness on explicit and implicit contributions to visuomotor adaptation over time. *Experimental Brain Research*, *236*(7), 2047–2059.

Oza, A., Kumar, A., Sharma, A., & Mutha, P. K. (2024). Limb-related sensory prediction errors and task-related performance errors facilitate human sensorimotor learning through separate mechanisms. PLoS Biology, 22(7), e3002703.

Taylor, J. A., Krakauer, J. W., & Ivry, R. B. (2014). Explicit and implicit contributions to learning in a sensorimotor adaptation task. The Journal of Neuroscience: The Official Journal of the Society for Neuroscience, 34(8), 3023–3032.

Tsay, J. S., Lee, A., Ivry, R. B., & Avraham, G. (2021). Moving outside the lab: The viability of conducting sensorimotor learning studies online. *Neurons, Behavior, Data Analysis, and Theory*, *5*(3). https://doi.org/10.51628/001c.26985

Tsay, J. S., Haith, A. M., Ivry, R. B., & Kim, H. E. (2022a). Interactions between sensory prediction error and task error during implicit motor learning. *PLoS Computational Biology*, *18*(3), e1010005.

Tsay, J. S., Kim, H., Haith, A. M., & Ivry, R. B. (2022b). Understanding implicit sensorimotor adaptation as a process of proprioceptive re-alignment. *eLife*, *11*.

Uehara, S., Mawase, F., & Celnik, P. (2018). Learning similar actions by reinforcement or sensory-prediction errors rely on distinct physiological mechanisms. *Cerebral cortex*, *28*(10), 3478-3490.

Reviewer #2: This manuscript reports six experiments that bolster a previously proposed perceptual prediction error (PPE) account of motor learning. The data are solid and the writing is clear. The replication across paradigms is valuable.
That said, I do not see a substantive new computational contribution beyond prior PPE work. More importantly, the paper assumes a division in which implicit learning is PPE-driven and explicit re-aiming is performance/task-error (PE)-driven, but it does not directly test or rule out the possibility that explicit learning also has a PPE component. For PLOS Computational Biology, where advances in computational concepts are expected, I am not inclined to recommend acceptance in the current form. The dataset itself is strong and may fit better at a venue that emphasizes rigorous empirical reports without a new modeling idea (e.g., Journal of Neurophysiology).

Thank you for recognizing our previous work on the PPE model for implicit adaptation. However, our previous study was only applied to a particular paradigm, i.e., the error-clamp learning with isolated implicit adaptation. Challenging two dominant theoretical frameworks (SPE- and PE-based models) with one particular paradigm is insufficient. The current study is a critical step to advance the field as it applies the same PPE framework to tackle a more widely-studied paradigm: the visuomotor rotation with task-relevant perturbations. More importantly, the VMR paradigm also invokes explicit learning, making it essential to examine whether the same PPE framework can parsimoniously explain a more common case in human learning, i.e., learning with both cognitive and procedural apparatus.

You constructively suggested some alternative error signals for explicit learning (based on PPE). We did not explore this possibility in our initial manuscript because the field unanimously agree that explicit learning is driven by PE (see prominent reviews by Krakauer et al., 2019 and Morehead & Orban, 2021), and we also concur with this idea. Our paper focuses on implicit learning, where the field currently has a heated debate. Nevertheless, as this is your major concern, we have now compared this “new” alternative model of explicit learning and showed that our data do not support PPE as a viable candidate for explicit learning. We believe this new modeling work makes the exploration of error signals for motor learning more complete.

**Major comments**
1. The paper interprets explicit as PE-driven and implicit as PPE-driven, but there is no head-to-head model test. Please add a formal comparison where the explicit state is driven by (i) PE only, (ii) PPE only, and (iii) PE+PPE, with AIC/BIC (or CV) and parameter plausibility. This will address whether explicit learning carries a PPE component.

We agree that a formal model comparison could strengthen our claims about the differential roles of PE and PPE in explicit and implicit learning. However, it is important to recognize that there is a consensus in the field that PE drives explicit learning. PPE has never been proposed as a driver of explicit learning, and we, as proponents of the PPE framework for implicit learning, do not endorse this idea either. There are compelling empirical reasons why PPE-driven explicit learning is implausible. First, PPE exhibits a nonlinear relationship with perturbation size, whereas explicit learning increases linearly with perturbation size, as widely shown in the literature (Bond & Taylor, 2015; McDougle & Taylor, 2019) and our present study. Without running formal modeling, we shall recognize that explicit learning is unlikely to be driven by PPE. Second, the magnitude of PPE is small (typically 5-7°), as shown by model predictions and direct measurements in our Exp 6. However, explicit learning can occur rapidly and reach large magnitudes under large perturbations. This mismatch also makes PPE an implausible candidate for driving explicit learning.

Nevertheless, to directly address this concern, we conducted a formal comparison of these three alternative models, where explicit learning is driven by: (i) PE only, (ii) PPE only, and (iii) PE and PPE concurrently. Implicit learning is driven by PPE in all these three models. The results showed that the two models with a PPE-driven explicit component failed miserably (Figure R1). For Experiment 1, the linear scaling of explicit learning as a function of perturbation size cannot be captured by PPE-only. Although it appears that “PE+PPE” can account for explicit learning, it produces erroneous predictions for implicit learning (Figure R1A). Only our original model, with PE-driven explicit learning and PPE-driven implicit learning, can parsimoniously explain both components of learning (details of model comparison in Table R1).

The same holds for Experiment 2. The PE-only model predicts that the one-step condition should yield larger implicit learning than the stepwise condition, which directly contradicts the empirical finding (Figure R1B). Although the PE+PPE model, which combines PE and PPE to drive explicit learning, appears capable of capturing the pattern in which implicit learning increases under the stepwise condition and exceeds that in the abrupt condition, the magnitude of the difference between the two conditions does not match the empirical data. And the “PE+PPE” model exhibits worse goodness of fit compared to the original PPE model (R2 = 0.935 vs. 0.960, BIC = 303.96 vs. 264.81, respectively).

In sum, using the large datasets from Experiments 1 and 2, we showed that PPE, either alone or combined with PE, cannot drive explicit learning. Our original model, with implicit learning driven by PPE and explicit learning driven by PE, remains the best model for empirical findings.

**Figure R1**. Model fits of the alternative models with PE-driven, PPE-driven or PE+PPE-driven explicit learning, using datasets from Experiments 1 and 2.

**Table R1**. Model comparisons (Exp. 1’s data)

|  | *R^2^* | *RMSE (°)* | AIC | BIC | ΔBIC | *w* |
| --- | --- | --- | --- | --- | --- | --- |
| **PE-only** | **0.969** | **4.807** | **361.67** | **375.27** | **/** | 1 |
| PPE-only | 0.422 | 16.143 | 633.05 | 646.65 | 271.38 | < .001 |
| PPE + PE | 0.963 | 5.436 | 389.23 | 402.83 | 27.56 | < .001 |

|  | *B_i_* | *A_i_* | *B_e_* | *A_e_* | *k* |
| --- | --- | --- | --- | --- | --- |
| **PE-only** | 0.151 | 0.981 | 0.513 | 0.981 | 0.204 |
| PPE-only | 0.800 | 0.701 | 0.999 | 0.701 | 0.100 |
| PPE + PE | 0.513 | 0.983 | 0.513 | 0.983 | 0.570 |

**Table R2**. Model comparisons (Exp. 2’s data)

|  | *R^2^* | *RMSE (°)* | AIC | BIC | ΔBIC | *w* |
| --- | --- | --- | --- | --- | --- | --- |
| **PE-only** | **0.960** | **3.965** | **252.43** | **264.81** | / | 1 |
| PPE-only | 0.469 | 12.30 | 451.65 | 464.04 | 199.23 | < .001 |
| PPE + PE | 0.935 | 4.952 | 291.57 | 303.96 | 39.15 | < .001 |

|  | *B_i_* | *A_i_* | *B_e_* | *A_e_* | *k_step_* |
| --- | --- | --- | --- | --- | --- |
| **PE-only** | 0.245 | 0.927 | 0.889 | 0.927 | 0.139 |
| PPE-only | 0.800 | 0.882 | 0.999 | 0.882 | 0.243 |
| PPE + PE | 0.475 | 0.868 | 0.682 | 0.868 | 0.177 |

2. The modeling framework looks very close to earlier PPE formulations. If there is a conceptual advance, make it explicit (state structure, error-to-state mapping, identifiable regimes, or new theory-level predictions). If not, please position the paper as a replication/validation study and adjust the claims accordingly.

We agree that our modeling framework builds upon our previous PPE formulations (Zhang et al., 2024). However, the present study represents critical conceptual advances and makes new scientific contributions that go beyond replication:

(1) Our previous work used the error-clamp paradigm, where visual feedback is task-irrelevant and participants were instructed to reach directly to the target. Thus, the error-clamp paradigm induces only implicit learning without any explicit strategy. However, most real-world and laboratory motor learning tasks involve both explicit and implicit processes. Our current study uses the classical visuomotor rotation paradigm, in which these two processes coexist, and offers a more generalizable and representative examination of motor learning. Critically, our current model offers a new insight into how explicit and implicit learning interact. The interaction of explicit and implicit learning is not a simple competition or compensation. Rather, explicit aiming, a manifestation of explicit learning, serves as an anchor (or a reference) for computing the perceptual prediction errors that drive implicit learning. This view echoes with the findings that implicit learning generalizes around the aiming direction, which is determined by explicit learning (Day et al., 2016; McDougle et al., 2017; Schween et al., 2018).
(2) The current VMR paradigm uniquely allows us to resolve the ongoing debate surrounding error signals for implicit motor learning. The error-clamp paradigm used in the previous study cannot disentangle performance error from prediction error. The error-clamp paradigm does not have an apparent performance error since participants are required to use the invisible hand to reach the target, and their performance error is unknown to them. In contrast, the conventional VMR paradigm allows us to objectively measure the performance error, i.e., the cursor deviation from the target. Moreover, in the error-clamp paradigm, the task goal and aiming direction are identical, making it impossible to determine which serves as the cue related to motor prediction. Using the VMR paradigm, we separate the target from the aiming direction (as participants need to re-aim away from the target), revealing that the prediction errors (both SPE and PPE) are computed relative to the aiming direction, not the task goal. Notably, this separation of PE and prediction errors plagued previous modeling works, including PEA (Zhang et al., 2024) or PReMo (Tsay et al., 2022) formulations. In sum, the VMR paradigm provides a critical testbed for comparing these competing error signals and establishing PPE as the primary driver of implicit learning.
(3) Other “minor” but important contributions: (i) explaining away the seminar findings from Mazzoni & Krakauer, 2006 study (our Experiment 4), i.e., an implicit drift of the hand despite its detrimental effect on performance. This implicit adaptation has been proposed to be driven by PE or SPE. We basically show that the implicit drift exhibits a previously unknown perturbation-size dependency that can only be explained by our PPE model. (ii) devising and verifying a method to measure PPE directly (our Experiment 6); and (iii) accounting for the increase in implicit learning under gradual perturbations (our Experiment 2), which has not been satisfactorily explained by previous studies.

We added more texts in the Discussion to emphasize these novel contributions in the section of “Theoretical advance of the PPE model relative to previous work” and “The relationship between explicit and implicit learning”. The distinction of aiming and movement goal, enabled by VMR, is emphasized in the Introduction.

3. Provide simulations (parameter recovery/confusion matrices) showing that your fitting pipeline can recover PPE-only, PE-only, and mixed drivers for explicit and implicit under realistic noise/sample sizes.

Basically, you requested a quality check for our model fitting. Indeed, these additional analyses can help examine the robustness of our model analyses. We performed parameter recovery and confusion matrix analyses and confirmed that our model fitting pipeline is robust. Results from these additional analyses are now included in the Supplemental Material in the revision.

We conducted parameter recovery analyses for the PPE model, using data from two representative experiments: Experiment 1, which represents the case where the model fits both explicit and implicit learning, and Experiment 4, which represents the case where the model fits only implicit learning, as explicit learning is constrained by re-aiming instructions. For each experiment, we employed a bootstrap resampling approach to estimate the parameter distributions from the empirical data. We then used the median ± standard deviation of the bootstrapped parameters to define realistic parameter ranges for simulation. Critically, we incorporated motor noise into the simulations, estimated from participants’ baseline movement variability (~1.5°), to ensure that our synthetic data reflected realistic noise characteristics.

For Experiment 1, the PPE model has five free parameters: Θ = [*k*, $A^{i}$, $B^{i}$, $A^{e}$, $B^{e}$]. We observed that the standard deviations of $A^{i}$ and $A^{e}$ were relatively small (~0.006), indicating small variability across participants, and that small variations in retention factors have minimal impact on the overall data patterns. Therefore, we focused our parameter recovery analysis on the three critical parameters: $B^{i}$, $B^{e}$, and *k*. The results showed great parameter recovery (Figure R2A). Simulated and recovered parameters showed high concordance, with concordance correlation coefficients (CCC) exceeding 0.98 for all three parameters.

For Experiment 4, the PPE model has three free parameters: Θ = [*k*, $A^{i}$, $B^{i}$]. We applied the same parameter recovery procedure. The results again showed strong concordance between simulated and recovered parameters (CCC > 0.7 for all parameters, Figure R2B). These results show that our fitting pipeline is reliable under realistic noise or sample sizes.

**Figure R2**. Results of parameter recovery analyses for the PPE model.

Following your suggestion, we also performed confusion matrix analyses, using datasets from Experiments 1 and 4. We generated synthetic data from each generative model and applied our model selection procedure (selecting the model with the lowest BIC) to these simulated datasets to assess whether the generative model could be correctly identified. The confusion matrices display the proportion of simulations in which each true generative model was recovered as the selected model.

The results show strong discriminability among the three alternative models (Figure R3). For Experiment 1, datasets generated from the PPE model were always best described by the PPE model, indicating perfect self-identification. The PE-generated datasets were correctly classified 75% of the time, with the remaining 25% spuriously favoring SPE, suggesting a moderate overlap between those two formulations. The SPE-generated datasets were recovered with high fidelity (0.98). For Experiment 4, the confusion matrix showed even stronger model identifiability, with perfect model recovery achieved across all three generative models. This indicates that under the specific design of Experiment 4, our fitting pipeline can unambiguously distinguish between the three models. Together, these results indicate that PPE is highly distinguishable from the alternative accounts, whereas the limited confusion between PE and SPE is unlikely to explain the model comparison results (the superiority of the PPE model) shown in our study.

**Figure R3**. Confusion matrices of the three models, for datasets from Experiments 1 and 4.

4. More direct intervention of PPE is necessary. Consider (or discuss) a manipulation that changes PPE while holding PE constant (e.g., altering visual/proprioceptive uncertainty or localization bias), and the converse. This would strengthen the separation claim.

Thank you for the constructive suggestion. We agree that direct interventions that manipulate PPE and subsequently affect implicit adaptation would provide strong support for our theory. We would like to clarify that our study actually employs multiple experimental manipulations to systematically alter PPE while holding PE relatively constant.

In Experiments 1, 3, and 4, we manipulated the perturbation size to alter PPE. PPE exhibited a nonlinear size-dependency, which reflected the influence of the changes in visual uncertainty, as predicted by the model feature that PPE is based on Bayesian estimation of the hand position. In Experiment 2, we manipulated how the perturbation was introduced. Compared to abruptly introducing a large perturbation, stepwise perturbation increased PPE (a natural consequence of the perturbation schedule), resulting in increased implicit adaptation. Importantly, the above manipulations changed PPE while holding PE constant. PE can be regulated by flexible explicit strategies, and as is evident in our data, participants across different conditions reached similar asymptotic performance levels during the plateau phase. Thus, PE remained relatively constant across conditions while PPE varied systematically.

Of course, more manipulations of PPE would further strengthen our claims. Theoretically, visual, proprioceptive, and motor prediction uncertainty can alter PPE without affecting PE. In our unpublished work, we manipulated proprioceptive uncertainty to directly alter PPE and found that the subsequent changes in implicit adaptation aligned with our model predictions. But with six experiments, one eye-tracking experiment, and some re-analyses of published data in the literature, we believe our study has provided sufficient evidence for our model in a single paper.

Minor comments

1. Define and use PPE, PE (task/performance error), SPE, and RPE consistently. A schematic (equations + flow/Vector diagram) contrasting these signals would help.

We schematically define and compare the three error signals in Figure 1 in our manuscript. In the revision, we make sure that PPE, PE, and SPE are used consistently as abbreviations.

2. Include full fit summaries (LL, AIC/BIC, ΔAIC/BIC, model weights), parameter CIs/posteriors, and basic diagnostics per experiment.

Thank you for your suggestion. In fact, we had provided goodness-of-fit results in the Appendix of the original manuscript, including R-square, RMSE, AIC, and BIC for each experiment. We have now added ΔBIC and model weights (*w*) to provide a more complete model comparison. We also added tables to represent the 95% confidence intervals for all parameter estimates (Tables S1-S6 in the Appendix). Additionally, we add a figure to visualize the estimated values of parameter *k* (the slope of visual uncertainty as a function of eccentricity) across different experiments (Figure R4; see below), which is a critical parameter of the PPE model. The figure shows the parameter estimated obtained through two fitting methods: bootstrap resampling (N = 5,000) and fitting the average learning data. The close alignment between these two approaches demonstrates the internal consistency and reliability of our model fitting procedure. As you suggested above, we also added simulation results for parameter recovery and confusion matrix. We believe these elaborate representations of modeling results can give readers a comprehensive picture of our model comparisons.

**Figure R4**. Estimated values of the parameter *k* (the slope of visual uncertainty as a function of visual eccentricity) of the PPE model across different experiments. Pink diamond markers indicate median values estimated using bootstrap resampling (N = 5,000), with horizontal error bars representing 95% confidence intervals. Purple line markers show parameter values estimated by fitting the average learning data, which closely align with the bootstrap median estimates, demonstrating robust and consistent model fitting across experiments.

**Table S1**. Model comparisons and parameters for the dataset from *Bond & Taylor, 2015*

|  | *R^2^* | *RMSE(°)* | AIC | BIC | *Δ BIC* | *w* |
| --- | --- | --- | --- | --- | --- | --- |
| **PPE Model** | **0.708** | **1.684** | **172.81** | **182.04** | / | 1 |
| PE Model | 0.177 | 2.188 | 254.54 | 260.69 | 78.65 | < .001 |
| SPE Model | -0.730 | 5.054 | 522.48 | 528.63 | 346.59 | < .001 |

|  | *B_i_* | *A_i_* | *k* |
| --- | --- | --- | --- |
| **PPE Model** | [0.117, 0.248] | [0.881, 0.955] | [0.162, 0.278] |
| PE Model | [0.048, 0.104] | [0.999, 0.999] | / |
| SPE Model | [0.011, 0.020] | [0.867, 0.942] | / |

*Model fits using a bootstrap resampling approach (N = 5,000)

**Table S2**. Model comparisons and parameters for the data from Experiment 1

|  | *R^2^* | *RMSE(°)* | AIC | BIC | *Δ BIC* | *w* |
| --- | --- | --- | --- | --- | --- | --- |
| **PPE Model** | **0.969** | **4.807** | **361.67** | **375.27** | / | 1 |
| PE Model | 0.957 | 5.870 | 404.43 | 415.31 | 40.04 | < .001 |
| SPE Model | 0.954 | 6.044 | 411.00 | 421.87 | 46.60 | <. 001 |

|  | *B_i_* | *A_i_* | *B_e_* | *A_e_* | *k* |
| --- | --- | --- | --- | --- | --- |
| **PPE Model** | [0.092, 0.255] | [0.968, 0.991] | [0.474, 0.558] | [0.968, 0.991] | [0.139, 0.301] |
| PE Model | [0.058, 0.081] | [0.979, 0.999] | [0.415, 0.491] | [0.979, 0.999] | / |
| SPE Model | [0.011, 0.016] | [0.965, 0.989] | [0.466, 0.549] | [0.965, 0.989] | / |

**Table S3**. Model comparisons and parameters for the data from Experiment 2

**Table S3a.** Fitting the one-step and stepwise groups together with a single set of parameters

|  | *R^2^* | *RMSE(°)* | AIC | BIC | *Δ BIC* | *w* |
| --- | --- | --- | --- | --- | --- | --- |
| **PPE Model** | **0.960** | **3.965** | **252.43** | **264.81** | / | 1 |
| PE Model | 0.933 | 5.245 | 299.68 | 309.59 | 44.78 | < .001 |
| SPE Model | 0.925 | 5.491 | 307.76 | 317.67 | 52.86 | < .001 |

|  | *B_i_* | *A_i_* | *B_e_* | *A_e_* | *k_step_* |
| --- | --- | --- | --- | --- | --- |
| **PPE Model** | [0.173, 0.331] | [0.915, 0.941] | [0.850, 0.926] | [0.915, 0.941] | [0.100, 0.182] |
| PE Model | [0.216, 0.315] | [0.946, 0.970] | [0.598, 0.698] | [0.946, 0.970] | / |
| SPE Model | [0.031, 0.063] | [0.889, 0.924] | [0.870, 0.942] | [0.889, 0.924] | / |

**Table S3b.** Assuming explicit learning changes across conditions, fitting the one-step and stepwise groups with separate explicit learning parameters

|  | *R^2^* | *RMSE(°)* | *AIC* | *BIC* | *Δ BIC* | *w* |
| --- | --- | --- | --- | --- | --- | --- |
| **PPE Model** | **0.962** | **3.854** | **251.42** | **268.76** | / | 0.998 |
| PE Model | 0.958 | 4.234 | 265.97 | 280.84 | 12.08 | 0.002 |
| SPE Model | 0.926 | 5.453 | 310.53 | 325.39 | 56.63 | < .001 |

|  |  |  | Stepwise | | | One-step | |
| --- | --- | --- | --- | --- | --- | --- | --- |
|  | *B_i_* | *A_i_* | *B_e_* | *A_e_* | *k_step_* | *B_i_* | *A_i_* |
| **PPE Model** | [0.167, 0.321] | [0.917, 0.944] | [0.494, 0.757] | [0.875, 0.965] | [0.100, 0.180] | [0.167, 0.321] | [0.917, 0.944] |
| PE Model | [0.181, 0.273] | [0.955, 0.984] | [0.162, 0.303] | [0.997, 0.999] | / | [0.181, 0.273] | [0.955, 0.984] |
| SPE Model | [0.032, 0.064] | [0.887, 0.919] | [0.556, 0.780] | [0.916, 0.971] | / | [0.032, 0.064] | [0.887, 0.919] |

**Table S3c.** Cross-validation: first fit the model by using the data from the stepwise group only, and then predict the data for the one-step group

|  | *R^2^* | *RMSE(°)* | *AIC* | *BIC* | *Δ BIC* | *w* |
| --- | --- | --- | --- | --- | --- | --- |
| **PPE Model** | **0.913** | **5.868** | **165.72** | **174.64** | / | 1 |
| PE Model | 0.661 | 9.031 | 201.66 | 208.80 | 34.16 | < .001 |
| SPE Model | 0.583 | 9.638 | 207.38 | 214.52 | 39.88 | < .001 |

|  | *B_i_* | *A_i_* | *B_e_* | *A_e_* |
| --- | --- | --- | --- | --- |
| **PPE Model** | [0.199, 0.430] | [0.885, 0.941] | [0.487, 0.812] | [0.885, 0.941] |
| PE Model | [0.282, 0.448] | [0.921, 0.977] | [0.363, 0.550] | [0.921, 0.977] |
| SPE Model | [0.072, 0.212] | [0.823, 0.904] | [0.616, 0.888] | [0.823, 0.904] |

**Table S4**. Model comparisons and parameters for the data from Experiment 3

**Table S4a.** For the trial-by-trial learning sequence (Figure 5B)

|  |  | *R^2^* | *RMSE(°)* | *AIC* | *BIC* | *Δ BIC* | *w* |
| --- | --- | --- | --- | --- | --- | --- | --- |
| **PPE Model** | **Imp** | **0.471** | **1.294** | **39.988** | **46.557** | / | 0.995 |
|  | Imp+Exp | 0.453 | 1.317 | 46.307 | 57.256 | 10.70 | 0.005 |
| PE Model | Imp | -0.682 | 1.747 | 77.622 | 82.001 | 35.44 | < .001 |
|  | Imp+Exp | -0.682 | 1.747 | 81.622 | 90.381 | 43.82 | < .001 |
| SPE Model | Imp | -0.682 | 1.747 | 77.622 | 82.001 | 35.44 | < .001 |
|  | Imp+Exp | -0.698 | 1.750 | 81.848 | 90.607 | 44.05 | < .001 |

|  | *B_i_* | *k* |
| --- | --- | --- |
| **PPE Model** | [0.338, 0.499] | [0.162, 0.228] |
| PE Model | [0.043, 0.065] | / |

**Table S5**. Model comparisons and parameters for the data from Experiment 4

|  | *R^2^* | *RMSE (°)* | *AIC* | *BIC* | *Δ BIC* | *w* |
| --- | --- | --- | --- | --- | --- | --- |
| **PPE Model** | **0.972** | **0.524** | **-32.81** | **-28.61** | / | 1 |
| PE Model | -0.866 | 4.496 | 94.19 | 97.00 | 125.61 | < .001 |
| SPE Model | -1.150 | 4.457 | 93.67 | 96.48 | 125.09 | < .001 |

|  | *B_i_* | *A_i_* | *k* |
| --- | --- | --- | --- |
| **PPE Model** | [0.164, 0.500] | [0.799, 0.892] | [0.118, 0.271] |
| PE Model | [0.027, 0.042] | [0.740, 0.835] | / |
| SPE Model | [0.020, 0.031] | [0.792, 0.866] | / |

**Table S6**. Model comparisons and parameters for the data from Experiment 5: Cross-validation (Fitting before-re-aiming phase, predicting after-re-aiming phase).

|  | *R^2^* | *RMSE (°)* | *AIC* | *BIC* | *Δ BIC* | *w* |
| --- | --- | --- | --- | --- | --- | --- |
| **PPE Model** | **0.882** | **5.704** | **156.26** | **164.94** | / | 0.332 |
| PE Model | 0.337 | 13.825 | 228.62 | 235.57 | 70.63 | < .001 |
| SPE Model | 0.859 | 5.843 | 156.59 | 163.54 | -1.40 | 0.668 |

|  | *B_i_* | *A_i_* | *B_e_* | *A_e_* | *k* |
| --- | --- | --- | --- | --- | --- |
| **PPE Model** | [0.084, 0.419] | [0.942, 0.964] | [0.289, 0.445] | [0.942, 0.964] | [0.140, 0.357] |
| PE Model | [0.065, 0.097] | [0.958, 0.974] | [0.230, 0.363] | [0.958, 0.974] | / |
| SPE Model | [0.013, 0.021] | [0.945, 0.963] | [0.272, 0.429] | [0.945, 0.963] | / |

3. Clarify what is replicated vs extended relative to explicit–implicit separation literature (aim reports, clamped feedback, localization shifts).

While our work adopts established explicit-implicit framework, it extends the theoretical understanding of how explicit and implicit components interact.

At the methodological level, we adopted established methods from the literature to measure and separate explicit and implicit learning components. Specifically, we used exclusion trials to measure implicit learning in each experiment, and we measured explicit learning through aiming reports in Experiments 5 and 6. In Experiments 5 and 6, we implemented a novel aiming report method: participants used their left hand to control a robotic arm to position a visual marker to indicate their aiming direction, Arguably, this approach provides a more precise measurement of aiming compared to the traditional methods used in previous studies, such as number dials (Taylor et al., 2014), positioning a visual marker by pressing the arrow keys (Miyamoto et al., 2020), or touchscreen pointing (Bond & Taylor, 2017). More importantly, Experiment 6 also required the participants to report their perceived hand position after a perturbed reaching movement, and we can directly estimate the size of PPE by comparing the two visual-mark-based reports before and after perturbation.

At the theoretical level, our PPE model provides a new perspective on how the two components interact. Rather than treating explicit and implicit learning as simply operating in parallel (Taylor et al., 2014; Bond & Taylor, 2015; McDougle et al., 2015), or competing for the same error signal (Albert et al., 2022), our model proposes a dynamic interaction: explicit aiming (as a result of explicit learning) serves as an anchor for PPE computation, which drives implicit learning.

Lastly, some studies found negative correlations between the explicit and implicit learning at the individual level, which has been interpreted as evidence that they compete (Albert et al., 2022; Maresch et al., 2021; Tsay et al., 2021), while some recent studies found a lack of correlation (D’Amario et al., 2024). However, our comprehensive analysis of the relevant datasets reveals that both patterns are possible: explicit and implicit learning do not consistently show negative correlations when measured independently and sometimes they show a lack of relationship (see Appendix Figure S8). We believe that elucidating their separate error signals, as in our PPE model, can help future investigations on the relationship of explicit and implicit learning.

**References**

Albert, S. T., Jang, J., Modchalingam, S., ’t Hart, B. M., Henriques, D., Lerner, G., Della-Maggiore, V., Haith, A. M., Krakauer, J. W., & Shadmehr, R. (2022). Competition between parallel sensorimotor learning systems. *eLife*, *11*.

Bond, K. M., & Taylor, J. A. (2015). Flexible explicit but rigid implicit learning in a visuomotor adaptation task. *Journal of Neurophysiology*, *113*(10), 3836–3849.

Bond, K. M., & Taylor, J. A. (2017). Structural learning in a visuomotor adaptation task is explicitly accessible. *Eneuro,* *4*(4), ENEURO.0122-17.2017.

D’Amario, S., Ruttle, J. E., ’t Hart, B. M., & Henriques, D. Y. P. (2024). Implicit adaptation is fast, robust and independent from explicit adaptation. In *bioRxiv*. <https://doi.org/10.1101/2024.04.10.588930>

Day, K. A., Roemmich, R. T., Taylor, J. A., & Bastian, A. J. (2016). Visuomotor learning generalizes around the intended movement. *eNeuro*, *3*(2), ENEURO.0005-16.2016.

Krakauer, J. W., Hadjiosif, A. M., Xu, J., Wong, A. L., & Haith, A. M. (2019). Motor learning. Comprehensive Physiology, 9(2), 613–663.

Maresch, J., Werner, S., & Donchin, O. (2021). Methods matter: Your measures of explicit and implicit processes in visuomotor adaptation affect your results. *The European Journal of Neuroscience*, *53*(2), 504–518.

McDougle, S. D., Bond, K. M., & Taylor, J. A. (2015). Explicit and implicit processes constitute the fast and slow processes of sensorimotor learning. *The Journal of Neuroscience: The Official Journal of the Society for Neuroscience*, *35*(26), 9568–9579.

McDougle, S. D., Bond, K. M., & Taylor, J. A. (2017). Implications of plan-based generalization in sensorimotor adaptation. *Journal of Neurophysiology*, *118*(1), 383–393.

McDougle, S. D., & Taylor, J. A. (2019). Dissociable cognitive strategies for sensorimotor learning. *Nature Communications*, *10*(1), 40.

Miyamoto, Y. R., Wang, S., & Smith, M. A. (2020). Implicit adaptation compensates for erratic explicit strategy in human motor learning. *Nature neuroscience, 23*(3), 443-455.

Morehead, J. R., & Orban de Xivry, J.-J. (2021). A synthesis of the many errors and learning processes of visuomotor adaptation. In *bioRxiv*.

Schween, R., Taylor, J. A., & Hegele, M. (2018). Plan-based generalization shapes local implicit adaptation to opposing visuomotor transformations. *Journal of Neurophysiology*, *120*(6), 2775–2787.

Taylor, J. A., Krakauer, J. W., & Ivry, R. B. (2014). Explicit and implicit contributions to learning in a sensorimotor adaptation task. *The Journal of Neuroscience: The Official Journal of the Society for Neuroscience*, *34*(8), 3023–3032.

Tsay, J. S., Lee, A., Ivry, R. B., & Avraham, G. (2021). Moving outside the lab: The viability of conducting sensorimotor learning studies online. *Neurons, Behavior, Data Analysis, and Theory*, *5*(3). https://doi.org/10.51628/001c.26985

Tsay, J. S., Kim, H., Haith, A. M., & Ivry, R. B. (2022). Understanding implicit sensorimotor adaptation as a process of proprioceptive re-alignment. *eLife*, *11*.

Zhang, Z., Wang, H., Zhang, T., Nie, Z., & Wei, K. (2024). Perceptual error based on Bayesian cue combination drives implicit motor adaptation. eLife, 13. https://doi.org/10.7554/eLife.94608

Reviewer #3: The authors build new theories, design experiments, and report data on how people adapt to visuomotor rotation. Amongst three models tested, a new “perceptual prediction error” model seems to capture elements of human motor adaptation. This work advances theoretical and experimental understanding of human motor adaptation; the importance of these advances are unclear, due to vagueness and internal contradictions in presentation, calculations, and interpretations.

Strengths:
The manuscript identifies gaps in explanations of human visual motor adaptation, when experiments and analyses separate performance into explicit and implicit components. Previously published work seems to indicate that steady state implicit learning shows either no dependence, or nonlinear dependence, on the strength of perturbation. The authors provide sufficient evidence that two extant theories fail to replicate this finding. A new theory posits that implicit adaptation arises from “perceptual prediction error,” (PPR) the difference between the aiming angle and the angle of the multisensory prediction of hand location. The authors draw from prior work, indicating visual uncertainty increases with perturbation angle. Theorized consequences of that result include Bayesian reweighing of multiple inputs, causing proprioception to increase its relative contribution as visual reliability decreases. From these considerations emerges an overall hypothesis: perceptual prediction errors, sensitive to increased visual noisiness and Bayesian reweighing, could drive implicit learning in visuomotor adaptation.

The manuscript provides solid theoretical bases for this construction, and provides several tests of the PPE model for implicit learning, against models driven by performance error (PE) and (visual) sensory prediction error (SPE). Several analyses indicate that the PPE model better predicts human adaptation, in asymptotic levels of implicit learning, dependence on perturbation strength, and trial-by-trial adaptation. PPE model performance is impressive in its mimicking of nonlinearity with respect to perturbation strength, in both overall implicit learning and trial-by-trial adaptation in the random environment.

The experimental testing of proprioceptive bias, through addition of a bimanual component, provides a particularly strong line of evidence for multisensory consequences of visuomotor rotation, irrespective of particular model choice.

The authors build a comprehensive case for their model, complete with analyzing previously published data; full reporting on their equations, computation methods, and fitted parameters; full online sharing of their code and results; and reference to appropriate literature. The authors provide insights into their reasoning, comparison to prior work, and opportunities for checks and balances through future studies.

Thanks for your fair and comprehensive summary of our study.

Weaknesses:
A major concern arises from the enlarged parameter and composite function space of PPE, compared with PE and SPE models. Analysis of the Bond & Taylor 2015 data generates state space model fits for PE and SPE solely from data, while PPE “as an additional four parameters” (lines 1221-1222). Whereas the rationale for this difference becomes apparent later in the manuscript, and some parameters are fixed from prior work, Figure 2 unfairly compares results arising primarily from data vs. deeper dependence on fits. This nuance is under-explained early in the manuscript and builds confusion for the reader.

Thank you for raising this concern about the fairness of model comparison given the different parameter spaces. We realized that our original presentation may have created confusion.

First, we’d like to clarify that the PPE model includes only one additional free parameter compared to the PE and SPE models, not four. Specifically, the additional free parameter is the slope pf visual uncertainty (*k*). The other three parameters related to sensory uncertainty ($\sigma_{u}$,$\sigma_{p}$, *b*) are fixed parameters derived from our previous work using the error-clamp paradigm (Zhang et al., 2024). These parameters represent inherent properties of the sensorimotor system that are relatively task-independent and thus could remain constant across perturbation experiments with center-out reaches. We left *k* as a free parameter because the slope of visual uncertainty could reasonably differ between error-clamp and visuomotor rotation paradigms due to their different aiming (or eye fixation) patterns. Importantly, even with one additional parameter, we properly account for model complexity by using AIC/BICs for model comparisons (shown in Tables in the Appendix). This ensures a fair comparison despite the difference in parameter space.

For model fits to the *Bond & Taylor, 2015* dataset, we recognize that Figure 2 may have created the misleading impression that PE and SPE models were fit differently than the PPE model. In fact, in Figure 2B, we presented empirical PE and SPE calculated directly from the data to illustrate the distinct temporal dynamics of these two error signals in this dataset. And, all three models were fit to the data using identical procedures. Specifically, we did not derive explicit learning by model fitting; for all the models, explicit learning was taken directly from the observed data. We fit only implicit learning data, which ensures that all models are evaluated on equal footing. We now stated this clearly in the Results. We also revised the related parts in the Methods to clarify these critical points.

A deeper problem arises from the Bayesian calculation of “perceived hand” built from combination of visual cue, proprioceptive cue, and the aim of the reach (line 1126). Aim is not a sensory input; it is a motor goal. While the statistical consequence is not equivalent, in spirit the authors seem to be confusing maximum likelihood and MAP calculations. At the very least, the authors need to justify the direct Bayesian combination of two sensory inputs with a motor goal.

It is confusing how this goal was estimated in trials where subjects did not explicitly report a goal. If the aim angle was estimated in these trials, then the authors have constructed a composite model, which conflates data and simulated inputs to subsequent simulations.

Thank you for raising this important conceptual point. We would like to clarify our rationale for including aiming as a cue in the Bayesian estimation of perceived hand position.

Aiming can serve as an internal predictive cue that is to be integrated with sensory feedback cues. We agree that aiming is not a sensory input in the traditional sense. Rather, it serves as an informative internal cue for estimating hand localization. Motor predictions, derived from efference copies or forward models of motor commands, serve as internal cues that are integrated with external sensory feedback to form state estimates. This perspective is well supported by theoretical and empirical works. We listed a few below:

Körding and Wolpert (2006) provide a comprehensive review outlining how the brain combines sensory inputs with efference copies of motor commands and prior knowledge to estimate states such as limb position amid noise. Within this Bayesian framework, the posterior estimate is computed as the product of the sensory likelihood and the motor prediction prior, weighted by their respective reliabilities. Wolpert (2007) further elaborate on this state estimate process through probabilistic sensorimotor models. They describe how forward models predict arm states from motor commands and are integrated with delayed sensory feedback via Kalman filter-like observers for real-time state estimation. This integration compensates for sensory delays and noise, yielding precise body state estimates that are superior to those based on sensory feedback alone. Specifically for hand pointing, studies by Saunders and colleagues (2004, 2015) also provided support for this multimodal integration, which suggested that participants continuously adjust pointing movements based on a state estimator that integrates visual feedback with efference copy predictions of hand position and proprioceptive cues. Their results matched predictions from Bayesian optimal weighting models, showing that motor predictions reduce reliance on noisier visual information during fast reaches. More recently, Laurens and Angelaki (2017) extended this framework to self-motion estimation, proposing a unified internal model that treats motor commands as a sensory “modality” input to a Bayesian Kalman filter alongside vestibular signals. Their simulations and neural data suggest that forward models transform efference copies into predicted inertial cues, which are optimally fused with sensory feedback. This integration explains phenomena such as reduced neural responses during active versus passive movements and accurate heading perception. Together, these studies establish that motor predictions can serve as fundamental internal cues that are optimally integrated with external sensory information to generate accurate and robust state estimates in sensorimotor control.

Similar modeling choices have been adopted in related works by other laboratories (e.g., Tsay et al., 2022) and our own lab (Zhang et al., 2024). In the revised manuscript, we now clearly state what this cue is with references when we first introduce the model:” Notably, motor prediction can not only provide a reference for error computation, but also serve as an internal predictive cue that is integrated with sensory feedback cues (34-37).”

For the VMR paradigm investigated here, aiming (motor prediction) occurs independently of explicit aiming reports. In other words, motor prediction does not depend on whether participants explicitly report their aim. For goal-directed movements, the motor system continuously generates predictions about the sensory outcomes of actions (Wolpert & Miall, 1996; Körding & Wolpert, 2006; Shadmehr & Krakauer, 2008; Wolpert, 2007). In our study, we estimated the aiming direction (as in Experiments 1 and 2), directly used observed aiming (as in Bond & Taylor, 2015), or used instructions to fix aiming (as in Experiments 4 and 5). Thus, our data and model comparisons are not limited by relying on fitting an unobserved aiming direction.

It is confusing how this model is proposed as a core learning rule, when “frequent aiming reports can alter the composition of explicit and implicit learning” (lines 250-251), suggesting deeper cognitive elements of learning than suggested here.

We double checked our manuscript to ensure that we have not over-claimed our model as a core learning rule, though we believe that elucidating the error signal for implicit learning is crucial for theorical advances in motor learning. On the other hand, we agree that various cognitive factors can affect motor learning, i.e., the composition (i.e., the relative contribution) of explicit and implicit learning, such as task settings, instructions, or aiming reports. However, these affecting factors do not take away the contribution of our current investigation, i.e., showing that PPE can outperform other candidate error signals for implicit learning in various task settings. This is precisely what our Experiment 1 addresses: no matter whether frequent aiming reports are used, the PPE model consistently outperformed the other two competing models.

More moderate concerns arise from main text that seems to cover many topics all at once, rather than concentrating on key concepts. Around Figures 2 and 3, individual paragraphs jump across many details of behavior and modeling, generating unclear emphases and conclusions. (A counterexample, featuring very clear organization, is the paragraph starting at line 312 which demonstrates the result and importance of proprioceptive biases.)

Thank you for this feedback. We have revised the presentation around Figures 2 and 3 to provide clearer organization and emphasis on the key points. For Figure 2, we added descriptive subheadings to highlight the core messages we aim to convey. Although the figure presents multiple behavioral and modeling results, we emphasize these key points: (1) implicit learning exhibits a nonlinear pattern varying with perturbation size; (2) the three candidate error signals (PPE, PE, and SPE) have distinct size-dependency and dynamics, which result in distinct model predictions for implicit learning; (3) PPE’s nonlinear pattern makes it uniquely capable of capturing the nonlinear characteristics of implicit learning. We added text to guide readers through these central messages more clearly.

For Figure 3, to streamline the focus on model comparison, we moved the results of total and explicit learning to the Appendix, as all three models successfully predict the total and explicit learning equally well (both scaled linearly with perturbation size). The figure and its related text focus exclusively on implicit learning, where the models make divergent predictions. We also added descriptive text to emphasize how the different error patterns lead to distinct behavioral predictions and how only the PPE model successfully captures the observed pattern. Thus, Figures 2 and 3 are now similar in terms of representation, reducing cognitive load for readers.

Figure 2 (revised). Behavioral and model fitting results from *Bond & Taylor, 2015*.

Figure 3 (revised). Results of Experiment 1.

Experiments 4 and 5 did add some additional data and comparisons, indicating PPE captured features of learning better than PE and SPE. The cognitive elements, however, provide additional layers of processing, that may partially invalidate motor processing purported to be non-cognitive. The interpretation of a new PE in Experiment 5 is particularly troubling and seems to violate the authors’ own definitions from Figure 1.

We’d like to clarify that the new PE formulation (PE_re-aiming_) is not our proposal. It was raised by proponents of the PE model (Albert et al., 2022, in particular) in an attempt to reconcile the original PE framework with the classical implicit drift findings in Mazzoni & Krakauer (2006). We included their idea in our model comparison to demonstrate that even with this modification, the PE model cannot account for this type of data. Being able to explain Mazzoni & Krakauer’s dataset is extremely important since this seminar study prompted researchers to consider SPE as the driving signal for implicit learning. However, we show that neither SPE nor PE can explain this implicit learning by revealing a novel perturbation size-dependency of this implicit drift in Experiment 5.

In the revision, to reduce potential confusion for readers, we now explicitly state that PE_re-aiming_ is a different formulation from the original definition of PE, and it was proposed by Albert et al. (2022) as an extension of the PE framework to explain the classical findings.

Experiment 6 is also validating, but renews the major concern noted above, regarding the estimated nature of aim direction in most of this modeling.

We have addressed this issue above. In Experiment 6, we directly and independently measured both aiming direction (through aiming reports) and perceived hand direction (through localization reports), and derived PPE as their difference. As reiterated earlier, the existence of motor predictions does not depend on whether they are explicitly reported. The explicit aiming reports in Experiment 6 simply provide a direct behavioral measure of these predictions, allowing us to test our model predictions. In trials where aiming is not explicitly reported (as in the other experiments), motor predictions still occur and contribute to state estimation. Just as no-feedback probe trials are used to measure implicit learning, aiming reports are used to measure explicit learning, which manifests as the intended or predictive state.

The eye fixation results were somewhat interesting on their own, but do not clearly serve as a “control” for the main paper or as a supplemental set of data. Instead, the fixation data raises concern that hypothesized changes in arm motor processing may instead be a consequence of simultaneous eye and arm control, biasing visual processing.

We don’t understand what specific hypothesized changes in arm motor processing you are referring to. But center-out reaching does not require sophisticated simultaneous eye and arm control. As our eye-tracking experiment has shown, the eye fixation for visuomotor rotation adaptation is relatively straightforward: the eye fixation remains largely on their intended or aiming direction during hand reaching. We also believe this fixation pattern remain largely unchanged across conditions. Thus, we don’t believe eye-arm coordination plays a large role in determining motor adaptation in this simple center-out settings.

However, our eye-fixation experiment is necessary since it provides solid empirical support for the premise of our PPE model: as the perturbation size increases, the visual cursor increasingly deviates from the aiming direction, leading to the increase of the related visual uncertainty. We notice that labeling this eye-tracking experiment as a “control” experiment might cause some confusion. We now refer to it as “Eye-tracking experiment” instead of a “control experiment’ in the manuscript.

A minor point: "Re-aiming" may be an appropriate term in the sub-literature, but here, "aiming" would work well. "Re-aiming" infers a mid-reach correction or cognitive override, such as instructed in Experiment 5.

Thank you for this great suggestion. We now replace “re-aiming” with “aiming” throughout most of the text, as “aiming” is more appropriate for describing the general strategic learning component or motor prediction. But we keep the term “re-aiming” specifically in the context of Experiments 4 and 5, because the two experiments were adopted from the classic instructed re-aiming paradigm, where participants were explicitly instructed to change their aiming direction from the original target. In this context, "re-aiming" serves a specific technical purpose by emphasizing that participants are not aiming at the original target direction, but rather at a distinct re-aiming target direction as instructed. Thus, we retained this term in these two experiments.

Conclusion:
Overall the authors have proposed interesting ideas, developed relevant experiments, and reported interesting data: the proprioceptive bias results seem to be notable regardless of model choice. There is good evidence that a multisensory signal is more likely to drive difference in implicit learning strength, dependent on perturbation size. The authors should more clearly indicate rationale behind the PPE computations, more clearly focus conclusions and discussions, and more clearly indicate the relevance of late experiments and supplementary material.

We believe that our revision, by following your constructive suggestions above, has been improved substantially.

**References**

Albert, S. T., Jang, J., Modchalingam, S., ’t Hart, B. M., Henriques, D., Lerner, G., Della-Maggiore, V., Haith, A. M., Krakauer, J. W., & Shadmehr, R. (2022). Competition between parallel sensorimotor learning systems. *eLife*, *11*. https://doi.org/10.7554/eLife.65361

Körding, K. P., & Wolpert, D. M. (2006). Bayesian decision theory in sensorimotor control. *Trends in Cognitive Sciences*, *10*(7), 319–326.

Laurens, J., & Angelaki, D. E. (2017). A unified internal model theory to resolve the paradox of active versus passive self-motion sensation. *eLife*, *6*(e28074). https://doi.org/10.7554/elife.28074

Mazzoni, P., & Krakauer, J. W. (2006). An implicit plan overrides an explicit strategy during visuomotor adaptation. The Journal of Neuroscience: The Official Journal of the Society for Neuroscience, 26(14), 3642–3645.

Sarlegna, F., Blouin, J., Vercher, J.-L., Bresciani, J.-P., Bourdin, C., & Gauthier, G. M. (2004). Online control of the direction of rapid reaching movements. *Experimental Brain Research*, *157*(4), 468–471.

Sarlegna, F. R., & Mutha, P. K. (2015). The influence of visual target information on the online control of movements. *Vision Research*, *110*(Pt B), 144–154.

Shadmehr, R., & Krakauer, J. W. (2008). A computational neuroanatomy for motor control. *Experimental Brain Research*, *185*(3), 359–381.

Tsay, J. S., Kim, H., Haith, A. M., & Ivry, R. B. (2022). Understanding implicit sensorimotor adaptation as a process of proprioceptive re-alignment. *eLife*, *11*.

Wolpert, D. M., & Miall, R. C. (1996). Forward models for physiological motor control. *Neural Networks: The Official Journal of the International Neural Network Society*, *9*(8), 1265–1279.

Wolpert, D. M. (2007). Probabilistic models in human sensorimotor control. *Human Movement Science*, *26*(4), 511–524.

Zhang, Z., Wang, H., Zhang, T., Nie, Z., & Wei, K. (2024). Perceptual error based on Bayesian cue combination drives implicit motor adaptation. eLife, 13. https://doi.org/10.7554/eLife.94608

**Have the authors made all data and (if applicable) computational code underlying the findings in their manuscript fully available?**
The [PLOS Data policy](https://track.editorialmanager.com/CL0/https:%2F%2Fjournals.plos.org%2Fploscompbiol%2Fs%2Fmaterials-and-software-sharing/1/010f019a552365af-8579bd84-7f02-4c3c-8b2c-4ed4a4ac108c-000000/vv-IPI7ocwYvFfvGbwfcCK6pEYcXGVnWWbY1Pih59f8=236) requires authors to make all data and code underlying the findings described in their manuscript fully available without restriction, with rare exception (please refer to the Data Availability Statement in the manuscript PDF file). The data and code should be provided as part of the manuscript or its supporting information, or deposited to a public repository. For example, in addition to summary statistics, the data points behind means, medians and variance measures should be available. If there are restrictions on publicly sharing data or code —e.g. participant privacy or use of data from a third party—those must be specified.

Reviewer #1: Yes

Reviewer #2: None

Reviewer #3: Yes
