# Supplementary material for: Perceptual prediction error supports implicit process in motor learning
Source: PLoS Comput Biol. 2026 Apr 15;22(4):e1014196. doi: 10.1371/journal.pcbi.1014196 (PMC13102310; doi:10.1371/journal.pcbi.1014196)
Supplement: Attachment — Submitted filename: Response_to_Reviewers_auresp_2.docx [file pcbi.1014196.s020.docx]

Reviewer #1: The authors have done an excellent job responding to my concerns. I think this is a rigorous and impactful contribution.

Thank you for your insightful and constructive comments that have helped us along the way.

Reviewer #3: The authors provide appropriate responses to reviewer concerns, through additional analyses and figures, and substantive edits to the text. I find these edits answer several of my identified weaknesses. In particular the authors better explain the additional parameters needed to estimate PPE, the efforts to minimize free parameters by relying on previously published values, and the rationale for including aiming direction as an input to Bayesian integration. Overall the manuscript provides evidence that a state space model using PPE to build implicit learning outperforms models using PE and SPE, in capturing the strength of implicit learning across perturbation strengths, training patterns, and additional tasks. Mid-training tests of proprioceptive bias provide additional confirmation that proprioception plays a role in implicit learning during a visuomotor rotation task.

Whereas the additional text, figures, and supplements do address reviewer comments, I worry that the manuscript is now very long. I will leave it to the editors and authors to discern how much content is needed to communicate the central scientific message.

Thank you for raising this valid concern regarding the manuscript’s length. To streamline the main text and keep the central scientific message clear, we have moved several sections to the Appendix, including: (1) The parameter recover and confusion matrix analyses demonstrating the reliability and robustness of our modeling results (Appendix Figures S4 and S5); (2) The two additional fitting approaches in Experiment 2 (Appendix Figure S6).

At this point I have one substantive concern. I ask the editors to determine whether the concern is meaningful, and if so, if the authors address the concern. While the PPE model captures changes in implicit learning across perturbation strengths and task demands, I have trouble seeing how the PPE signal constitutes an “error” that drives learning within a training session.

- The dynamics of PPE do not seem appropriate for a learning process. The authors indicate throughout the persistent values of PPE, remaining quite flat throughout training (e.g. Figures 2B and 3B). This time series does not mimic the asymptotic curves traced by implicit learning. The PPE time series that are less flat seem even less related to learning. Initial simulations reveal a time change in early training (Figure 1B). Sizeable stepwise implicit learning could either accompany slopes in PPE (Figure 4B, between trials 7 and 15) or flat PPE (between trials 23 and 39). I acknowledge that the state space models generate quality fits, but it is difficult to process how PPE could be used as a “error signal” to drive these time series of learning.

- The magnitude of PPE does not seem appropriate for the cumulative learning. Throughout the manuscript PPE size is smaller than the accumulated implicit learning. For example, in Figure 3, PPE ranges from -6 to -3 degrees, but modeled implicit learning learning ranges from (roughly) 7 t0 14 degrees. This amplification does not seem plausible, either from casting PPE as an error signal, or from the state space equations with the listed solutions for A and B.

My current conclusion is that proprioception likely plays a role in implicit learning magnitude, and multisensory integration likely serves as a driver of changes in implicit learning across the experimental conditions. I am unclear why or how, however, this construction of PPE can serve as an “error signal” that drives learning, in an abstract sense or as simulated in these state space models.

We sincerely appreciate your careful and thoughtful evaluation of our framework. Your concern centers on two issues that may appear unintuitive within the state-space formulation: (1) how a persistent and relatively small error signal (PPE) can give rise to comparatively large accumulated implicit learning, and (2) how a temporally flat PPE time course can produce an asymptotic learning trajectory. Below, we clarify why both behaviors are natural consequences of standard state-space learning dynamics.

(1) Apparent “amplification” of learning by a small error signal

The observation that accumulated implicit learning exceeds the magnitude of the driving error is not unique to the present model; rather, it is a well-established empirical property of implicit adaptation. For example, in the error-clamp paradigm—commonly used to isolate implicit learning—small visual clamps (e.g., 2° or 5°) can induce large implicit shifts, often exceeding 20°. This phenomenon has been described as overcompensation (e.g., Morehead et al., 2017; Tsay et al., 2022; Zhang et al., 2024).

State-space models provide a straightforward mechanistic explanation for this effect. In a standard formulation,

$$x_{n+1}=A{\cdot x}_{n}+B\cdot PPE$$

learning on each trial is driven by the term ($B\cdot PPE$), while forgetting (or state decay) is governed by ($A$). If the error signal persists across trials—as is the case for PPE in our experiments—then the incremental error-based updates accumulate over time. Growth of the state continues until the trial-by-trial learning term is balanced by the decay term. At asymptote ($x^{*}$), this balance satisfies:

$$(1-A)x^{*}=B\cdot PPE$$

yielding

$$x^{*}=\frac{B}{1-A}\cdot PPE$$

Because (A) is typically close to 1 in implicit adaptation (i.e., strong retention), the factor $\frac{B}{1-A}$ is thus large and can substantially amplify the steady-state magnitude of PPE. Thus, the asymptotic learning magnitude exceeding the instantaneous PPE is not anomalous, but a mathematically expected property of the system. Importantly, large asymptotic values emerge not from a large single-trial correction, but from persistent accumulation under high retention.

(2) Distinct temporal dynamics of PPE and implicit learning

The difference between the time course of PPE (often step-like and relatively flat) and the gradual asymptotic trajectory of implicit learning is also a feature of state-space dynamics. In our data, PPE resembles a sustained input signal, whereas learning evolves gradually because the update parameter (B) is small (e.g., as estimated in Experiment 1). A small learning rate ensures that even a constant input produces a slow, exponential approach to asymptote.

More generally, the dissociation between fast error dynamics and slower state changes reflects the separation between input and state in linear dynamical systems. A constant or step-like error signal naturally generates an exponential learning curve when filtered through a system with high retention and low learning gain. Therefore, similarity between the time course of error and learning is not required for the error to function as a valid driver of adaptation.

We provide the full model implementation to allow transparent inspection and simulation of these dynamics. To our knowledge, most computational accounts of implicit adaptation—including those based on population coding—reduce to dynamical systems that are formally equivalent to state-space models. These models have consistently demonstrated robustness in capturing the characteristic accumulation and asymptotic behavior of implicit error-based learning.

We hope this clarification resolves the apparent inconsistency and makes explicit how PPE, despite its relatively flat temporal profile and modest magnitude, can serve as a principled driver of implicit adaptation within the state-space framework.

**References**

Morehead, J. R., Taylor, J. A., Parvin, D. E., & Ivry, R. B. (2017). Characteristics of implicit sensorimotor adaptation revealed by task-irrelevant clamped feedback. *Journal of Cognitive Neuroscience*, *29*(6), 1061–1074.

Tsay, J. S., Kim, H., Haith, A. M., & Ivry, R. B. (2022). Understanding implicit sensorimotor adaptation as a process of proprioceptive re-alignment. *eLife*, *11*.

Zhang, Z., Wang, H., Zhang, T., Nie, Z., & Wei, K. (2024). Perceptual error based on Bayesian cue combination drives implicit motor adaptation. *eLife*, *13*.
